# Cognitive effort for self, strangers, and charities

**DOI:** 10.1038/s41598-022-19163-y

**Published:** 2022-09-02

**Authors:** Gregory J. Depow, Hause Lin, Michael Inzlicht

**Affiliations:** 1 Department of Psychology, University of Toronto, 1265 Military Trail, Scarborough, ON M1C 1A4 Canada; 2 Hill/Levene Schools of Business, University of Regina, Regina, Canada; 3 Sloan School of Management, Massachusetts Institute of Technology, Cambridge, USA; 4 Rotman School of Management, University of Toronto, Toronto, Canada

**Keywords:** Neuroscience, Psychology

## Abstract

Effort is aversive and often avoided, even when earning benefits for oneself. Yet, people sometimes work hard for others. How do people decide who is worth their effort? Prior work shows people avoid physical effort for strangers relative to themselves, but invest more physical effort for charity. Here, we find that people avoid cognitive effort for others relative to themselves, even when the cause is a personally meaningful charity. In two studies, participants repeatedly decided whether to invest cognitive effort to gain financial rewards for themselves and others. In Study 1, participants (N = 51; 150 choices) were less willing to invest cognitive effort for a charity than themselves. In Study 2, participants (N = 47; 225 choices) were more willing to work cognitively for a charity than an intragroup stranger, but again preferred cognitive exertion that benefited themselves. Computational modeling suggests that, unlike prior physical effort findings, cognitive effort discounted the subjective value of rewards linearly. Exploratory machine learning analyses suggest that people who represented others more similarly to themselves were more willing to invest effort on their behalf, opening up new avenues for future research.

## Introduction

Effort is aversive^1^ and often avoided. In the lab, people will even endure physical pain to avoid cognitive effort^2^. However, in daily life people sometimes exert effort to help others for no obvious gain to themselves^3^. How do people decide *who* and *when* to help when effort is required?

Prosocial behaviour refers to voluntary individual behaviour intended to improve the well-being of others^4^. Engaging in prosocial behaviour is associated with a boost to well-being for the actor^5^, and the tendency to act prosocially is associated with social benefits such as increased friendship quality^6^ and romantic relationship formation^7^. However, it is also costly to engage in prosocial behaviours^8^, and these costs have often been operationalized in experimental and observational studies with the investment of time or money. As costs increase, people become less willing to make the decision to help^9^. Importantly, many prosocial behaviours in the real world require people to invest—not time or money—but effort.

Although investing effort to help others is a recurring problem for any social organism, past research has mainly focused on understanding the investment of effort for personal gain. Faced with two equally rewarding outcomes, humans tend to follow the ‘law of least work’, taking the path of action requiring less effort^10,11^. Faced with unequal rewards, one might expect individuals to select the option with the highest payout. However, the subjective value of rewards is not determined by the magnitude of the reward alone. Rather, the subjective value of a reward is discounted by the physical^12^ or cognitive^13,14^ effort required to obtain it. The shape of the effort discounting curve can be characterized through participants’ revealed preferences on repeated choices between a low effort and low reward option versus an effortful option with higher levels of effort and reward^15^. The situation is further complicated when the effort is prosocial—when the fruits of one’s labour will be enjoyed by another.

Not only do people avoid effort for their own benefit, but nascent work suggests they avoid it even more rigidly for others. In fact, people often display prosocial apathy, foregoing rewards for others to avoid exerting physical effort themselves^16^. Relative to young adults, older adults are more willing to invest physical effort for others, but still discount rewards more when investing effort for others relative to themselves^17^. On the other hand, cross-sectional (i.e., between-subjects) experimental work suggests that individuals are sometimes willing to exert greater physical effort to earn rewards for a charity than they will to earn rewards for themselves^18^. Thus, it remains unclear from past work whether individuals exhibit prosocial apathy broadly, or only for strangers.

Furthermore, in our technologically advanced society the effort required to help others is often cognitive, but it is not yet settled whether cognitive effort causes reward discounting in the same way as physical effort^15,19–21^. In addition, it is not clear whether the prosocial apathy behaviour that has been observed for physical effort^16^ will generalize to effort that is merely cognitive^22^. Thus, we developed an easily adaptable task to investigate and compare decisions to invest cognitive effort for the self and others. Here, we investigated willingness to invest cognitive effort for the self, a charity, and an intragroup stranger.

While rewards and effort costs play crucial roles in people’s decisions to invest prosocial effort^16^, these decisions are also shaped by people’s social preferences^23^, which are in turn influenced by factors such as empathy and self-other overlap^24,25^. Empathy is a multidimensional process of understanding, sharing, and caring about the emotions of others^3,26^, while self-other overlap is a property of one persons’ perception of another person that includes perceived closeness between self and other, as well as the extent to which representations of self and other overlap^24,27^.

Empathy is a strong driver of helping others for no apparent gain to the self^28,29^, but some argue this relationship is explained by perceived self-other overlap^30^ and that empathy and self-other overlap are tightly related^31^. Although empathy is clearly a driver of prosociality^25,32^, it is also biased and parochial^33^, meaning individuals empathize more often, and to a greater extent, with close others^3^. Therefore, empathy may not reliably promote prosocial effort across social contexts^34,35^. Further, empathy itself requires effort and motivation^36,37^.

While self-other overlap is often measured with a single self-report item^24^, factor analyses suggest it is actually a multidimensional construct. Specifically, self-other overlap involves perceived closeness, or the closeness of the relationship between self and other, and overlapping representations, or the extent to which ones representation of self is overlapping with their representation of the other^38^. Recent work has found that overlapping representations track social closeness^27^, and correlate with real-world prosociality such that those who have donated an organ to a stranger show greater overlap in self and other representations^39^. That is, extraordinary altruists who volunteered to donate an organ to a stranger actually show greater overlap in their neural representations of self and strangers.

Here, we explored a new measure of overlapping representations derived from multivariate analysis, a popular neuroimaging technique used to investigate how information is encoded in patterns of activity across many brain voxels^40–42^. Whereas neuroimaging multivariate pattern analyses jointly analyze multi-voxel data to predict or decode stimuli or mental states, our multivariate decoding approach used only behavioral indices (e.g., task performance, reaction time) to decode stimuli and represent mental states that may be associated with overlapping representations^42^. Specifically, we examined whether individuals with highly overlapping representations of self and other—such that machine learning classifiers struggle to distinguish between self and other trials—may be more likely to engage in prosocial effort for others.

## Current study

**Figure 1:**
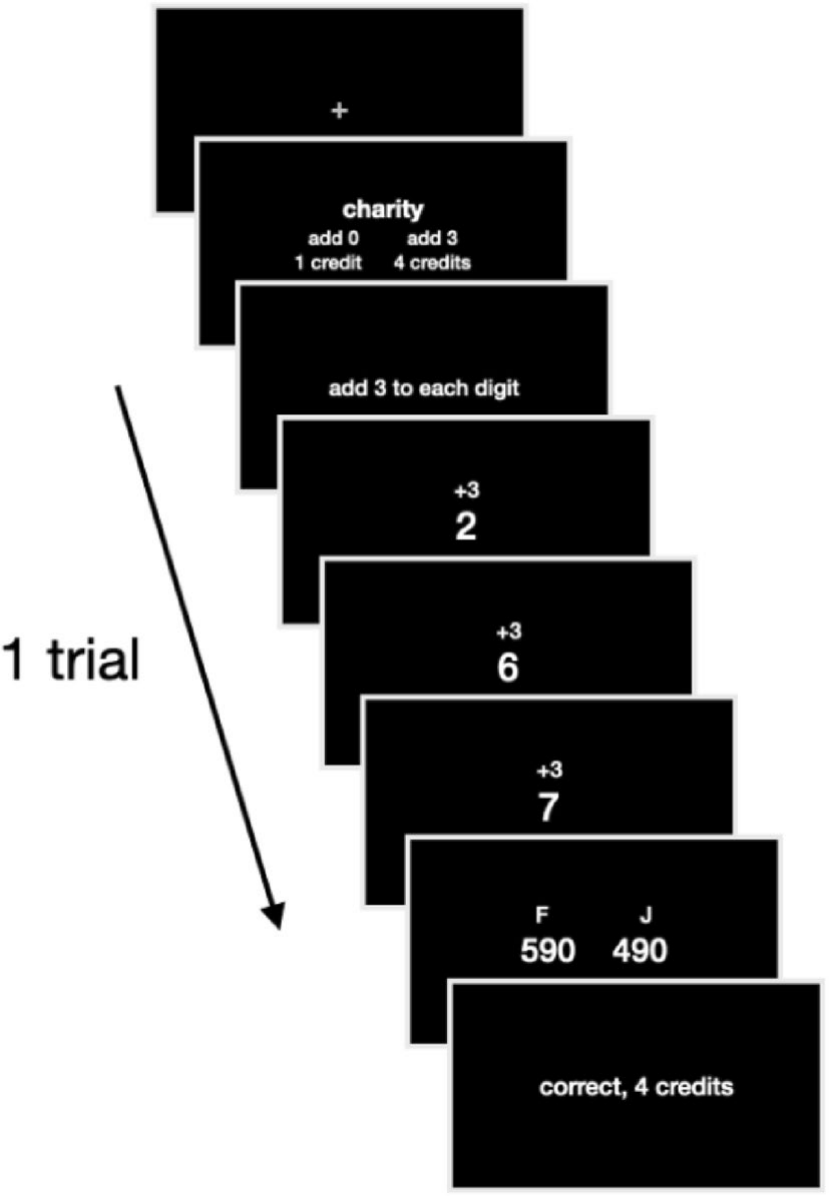
Procedure of a single trial. On each trial, the target (self, charity, or intragroup stranger) was shown at the top. Participants had 5 s to choose between a baseline option (fixed effort and reward) and a more effortful and higher reward option (effort and rewards varied across trials). They then performed their chosen task (add a number to three digits), indicated the correct response (3 s deadline), and saw feedback about their choice. Each digit appeared for 0.50 s, with a 0.70 s blank interval between digits, and a 0.50 s blank interval after all digits had been shown. Participants made 75 choices per target. Credits earned were converted to real money at the close of the experiment.

In two preregistered studies (osf.io/rc4an; osf.io/ncs97), participants repeatedly decided whether to exert cognitive effort to earn additional monetary rewards. On some trials they worked for themselves, but on other trials they made decisions for a preferred charity or an intragroup stranger (i.e., another unknown student; Fig. 1).

We analyzed choices with generalized mixed-effects models to test whether participants exhibit prosocial apathy when cognitive effort is required. Next, we fitted and compared models to examine the form of cognitive effort-discounting. We then trained machine learning classifiers on multivariate data to decode between self and other trials, which allowed us to explore the relationship between overlap of self-other representations and prosocial effort.

## Results

**Figure 2:**
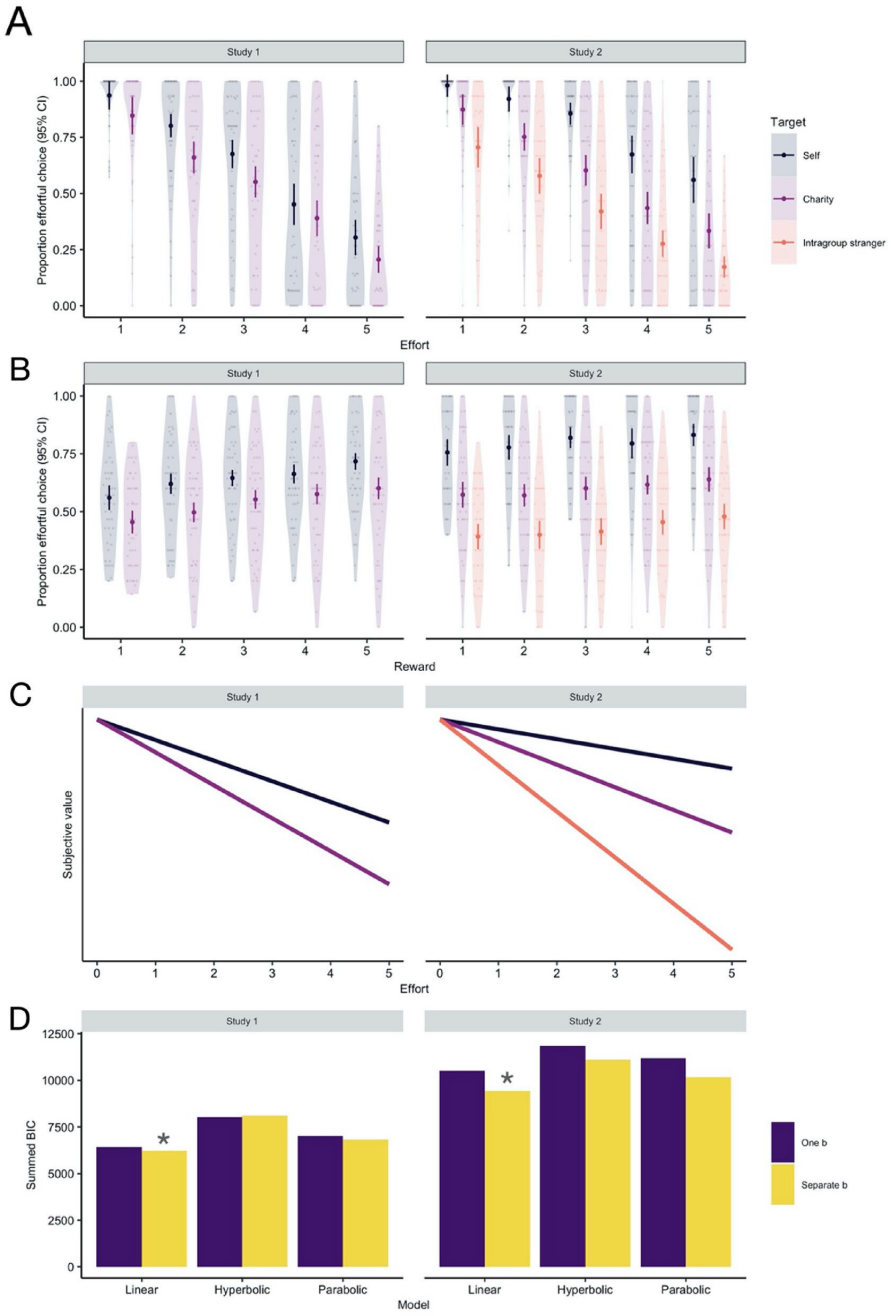
Proportion of trials where participants chose the effortful option (95% CI) rather than the baseline option at different effort levels (**A**) and reward levels (**B**) when working for self, charity, and intragroup stranger. Rewards were discounted by cognitive effort by different amounts for self and others. A linear discounting model with separate *k* discounting and *b* softmax parameters (**C**) fitted data best and had the smallest summed Bayesian Information Criteria (BIC) values (**D**).

Participants chose the effortful option less frequently when more effort was required (Fig. 2A; study 1: z = − 28.01, p < 0.001, r = − 0.50; study 2: z = − 19.89, p < 0.001, r = − 0.46), but more frequently when reward was higher (Fig. 2B; study 1: z = 7.86, p < 0.001, r = 0.14; study 2: z = 3.56, p < 0.001, r = 0.09). The effect of reward, though clearly significant in both studies, was about 4–5 times smaller than that of effort.

Importantly, people were cognitive misers for others, meaning they often passed up rewards for others to avoid mental effort themselves. Regardless of effort and reward, participants chose the effortful option less when working for their preferred charity than for themselves (study 1: z = − 11.28, p < 0.001, r = − 0.19; study 2: z = − 21.36, p < 0.001, r = − 0.43), and less often still for a stranger relative to themselves (study 2: z = − 31.58, p < 0.001, r = − 0.58). Participants also chose to work for an intragroup stranger significantly less often than for their preferred charity (study 2: z = − 14.55, p < 0.001, r = − 0.23).

Participants' choices were best described by a model where rewards were discounted by effort in a linear fashion (Fig. 2C), $subjective \, value=reward\times (1-{k}_{target}\times effort)$see Ref.^16^, with target-specific discounting (${k}_{target}$) and softmax (${\beta }_{target}$) parameters (Fig. 2D). Thus, whereas physical effort discounts rewards parabolically, our results suggest cognitive effort discounts rewards in a linear fashion, at least in our paradigm. As models with the lowest BIC may nonetheless be poor models of behavior^43–45^, we ran parameter recovery to assess model fit. We found model-predicted choices were highly correlated with observed choices (all r’s > 0.96). For full results see Online SM (Figs. S7–S10; Tables S12–S15).

**Figure 3:**
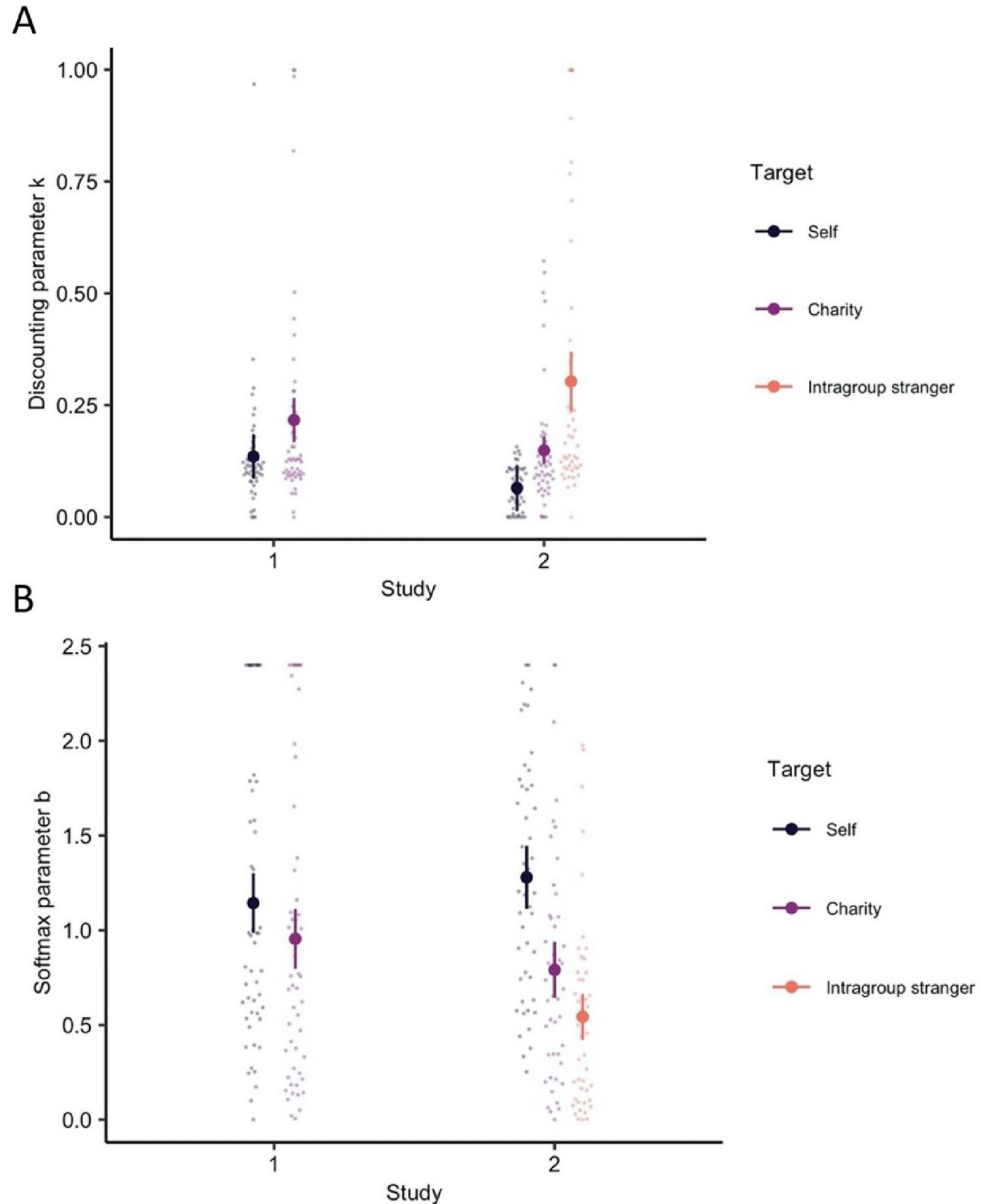
Discounting parameter *k* from the winning computational model (**A**). Higher *k* discounting parameters indicate that the subjective value of rewards was discounted by effort more steeply when working for others relative to the self. Softmax parameter *b* (**B**) from the same model. Lower *b* softmax parameters indicate participants’ choices were less consistent when working for others. Error bars are 95% CI.

Relative to themselves, participants discounted rewards more for charity (i.e., larger $k$ parameters; study 1: r = 0.20, p = 0.039; study 2: r = 0.24, p = 0.023), and for an intragroup stranger (study 2: r = 0.57, p < 0.001). They also discounted more for the stranger than charity (study 2: r = 0.41, p < 0.001; Fig. 3A). Interestingly, the softmax parameter $\beta$ was smaller when choosing for others relative to self, indicating participants’ choices were less consistent when working for others (Fig. 3B).

Participants’ motivation to invest effort might be driven not only by demands of the current task, but also by their level of fatigue at the time of decision^46–48^. Participants may become less willing to invest effort^49^ and shift their priorities to invest effort for self and others^50,51^ as time on task increases. We therefore performed exploratory analysis to examine how effortful choices for self and others changed over the course of the study. In Study 1, trial number was not significantly associated with choice (b =  − 0.07, SE = 0.05, z = − 1.47, p = 0.143), but it interacted with effort level (b = − 4.39, SE = 0.85, z = − 5.18, p < 0.001): As trial number increased, participants avoided choosing more effortful options even more (see Online SM, Fig. S20). In Study 2, participants chose the effortful option less across trials (b = − 0.117, SE = 0.05, z = − 3.53, p < 0.001), and trial number interacted with target (Fig. S21). Specifically, relative to when choosing for themselves, participants became less likely to choose the effortful option when the target was charity (b = − 1.89, SE = 0.62, z = − 3.02, p = 0.003) but not intragroup stranger (b = − 1.16, SE = 0.70, z = − 1.65, p = 0.099) over time. Overall, participants became less willing to invest effort in certain conditions over time. However, the exploratory nature of these analyses and the inconsistency of the results across studies suggests caution in interpreting the results.

When investing effort for others, motivation may also depend on social factors, such as the extent to which self and other representations overlap^39^. To quantify overlapping self-other representations, we trained machine learning classifiers (linear support vector machine; SVM) separately on each participant’s data to decode whether the target on each trial was self or other, i.e. for a charity or intragroup stranger. The classifiers were trained on five features: choice decision time, task accuracy, task reaction time, effort, and reward (choice was omitted as a feature to avoid introducing potential circularities with subsequent analyses). Classification accuracies will be near chance level (50%) if the multivariate representations of self and other trials are largely overlapping, whereas higher classification accuracies indicate less representational overlap.

Classification accuracies were 5.54% higher when the models were classifying whether the target was intragroup stranger (or self; 59.35%) than when the target was charity (or self; 53.81%) (b = 5.54, SE = 1.27, t(45) = 4.36, p < 0.001, r = 0.54), suggesting less overlapping multivariate self-target than self-charity representations. In other words, self-charity representations were more similar, which might be unsurprising since participants chose a personally meaningful charity to support. These findings are also congruent with the behavioral results, where participants were more willing to invest effort for charity than for an intragroup stranger.

**Figure 4:**
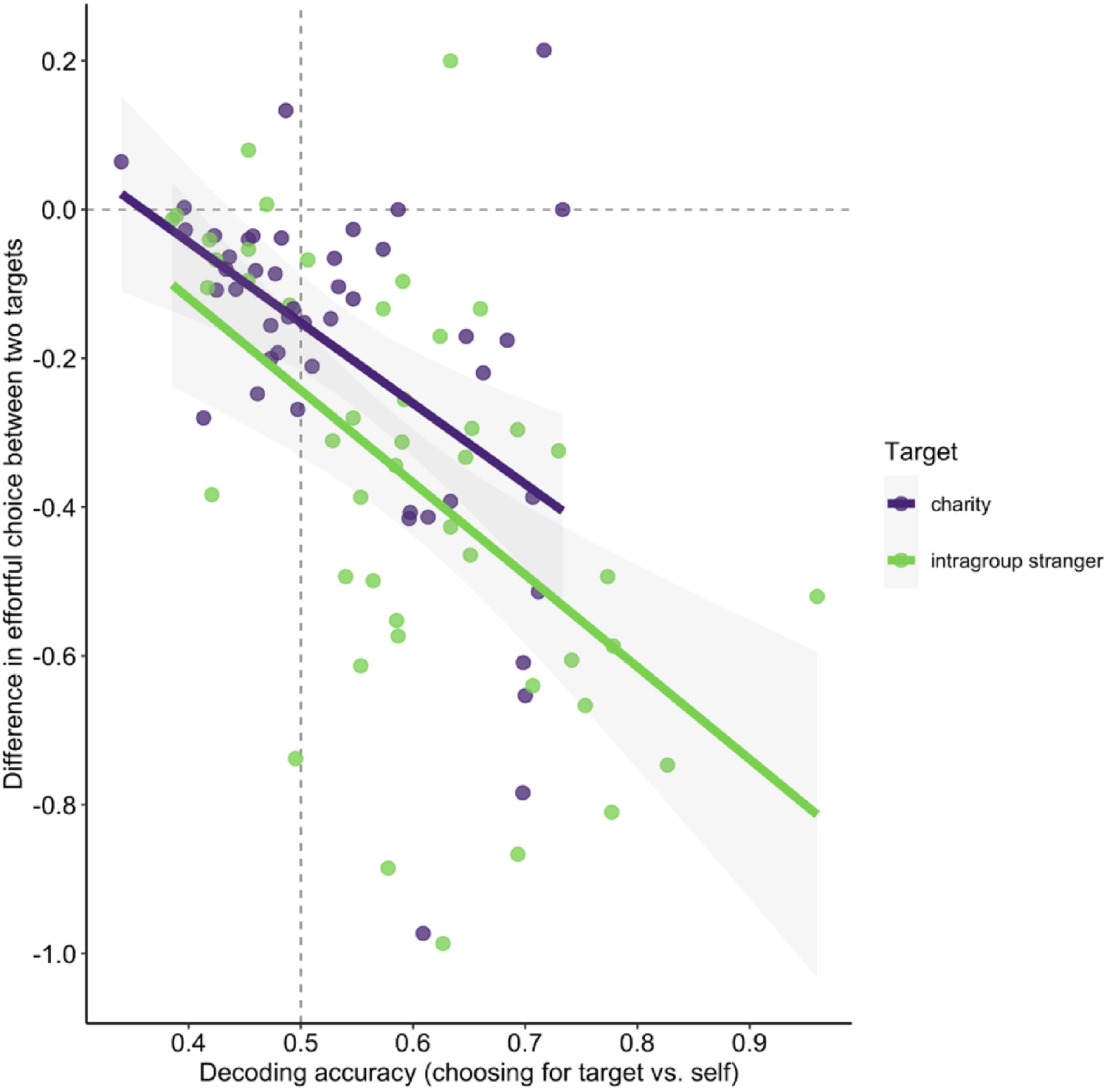
Difference in effortful choice between self and other trials as a function of linear support vector machine decoding or classification accuracy. Dots are individual participants’ data for charity (purple) and stranger (green) trials. Dots below or above the dashed horizontal line are participants who chose the effortful option less or more, respectively, for the other target relative to themselves. The vertical dashed line indicates chance-level classification accuracy (50%) when decoding whether the target on any given trial was self or other target (charity or stranger). Higher classification accuracies indicate less representational overlap and are associated with choosing the effortful option less for others.

We also observed substantial individual differences in classification/decoding accuracies (Fig. 4) that might reflect psychologically meaningful differences in representational overlap. That is, when accuracy is high, representational overlap is low, suggesting little self-other overlap, and thus participants should be *less* willing to exert effort for others. Conversely, when accuracy is near chance level (50%), representational overlap is high, suggesting greater self-other overlap, and thus participants should be *more* willing to exert effort for others.

Consistent with the above, classification decoding accuracies for charity (vs. self) was negatively correlated with prosocial effort for self relative to charity (b = − 1.08, SE = 0.29, t(44) = − 3.72, p < 0.001, r = − 0.49; Fig. 4). Similarly, classification accuracies for intragroup strangers (vs. self) was negatively correlated with prosocial effort for self relative to stranger (b = − 1.24, SE = 0.28, t(44) = − 4.43, p < 0.001, r = − 0.56; Fig. 4). These results suggest that participants whose representations of self overlapped more with their representations of stranger or charity were more willing to invest mental effort on their behalf.

Further analyses highlight the robustness of these results: When we fitted the models to data from all participants (ignoring preregistered exclusion criteria) or excluded choice decision time (to further eliminate potential circularity), we found the same pattern of results (Online SM, Fig. S11). Similarly, when we trained linear SVMs to instead decode the amount of effort or reward on each trial, we also found the same pattern of results whereby greater representational overlap was associated with increased willingness to exert effort for others (Online SM, Fig. S12).

In exploratory analysis, we tested whether accuracy at decoding self from other trials would be associated with the compassion aspect of the agreeableness factor from the Big 5^52^. We hypothesized that participants high in compassion would show greater overlap between their self-representations and their representations of strangers and charities. Thus, we expected to see reduced classification accuracy for target as compassion increased. Results supported these predictions. Compassion was negatively correlated with classification accuracy for charity, b = − 0.08, SE = 0.02, t(44) = − 4.07, p < 0.001, r = − 0.52, and for stranger, b = − 0.07, SE = 0.03, t(44) = − 2.41, p = 0.020, r = − 0.34; Online SM, Fig. S18. In other words, participants with high overlap between their representations of self and others were more compassionate, and more willing to invest mental effort for the benefit of charities and strangers.

## Discussion

Cognitive effort causes people to discount rewards not just for themselves but also for a personally meaningful charity and a stranger. In fact, the subjective value of financial rewards declined more steeply as effort rose on trials where rewards would accrue to a target other than the self. These findings suggest that people exhibit prosocial apathy not only for physical effort^16^, but also for cognitive effort: People will forgo financial rewards for others to avoid cognitive work themselves. Nonetheless, we know humans are a particularly altruistic species^53^ that often engages in prosocial behaviour in daily life^3^, so it is unsurprising to observe causal heterogeneity^54^ in our results. That is, individuals varied in their willingness to engage in prosocial effort, especially for intragroup strangers.

Our multivariate analyses provide preliminary insights into how people decide for whom they are willing to exert cognitive effort. Overall, participants showed greater representational overlap and greater effort for charities than strangers. In addition, we saw main effects of self-other overlap for both targets. Participants whose self-representation overlapped with their representation of charity more highly invested more effort on behalf of the charity. Finally, participants whose representations of intragroup strangers were more similar to their representations of themselves were more also willing to help strangers. These results suggest that when making prosocial cognitive effort decisions, people are not homo economicus only considering potential costs and rewards. Instead, social considerations like how similar they think others are to themselves^38,39^ and how their actions might impact others^55,56^ also influence decisions. The multivariate approach we take here to measuring extent of overlap between self and other representations provides a simple yet flexible framework for integrating behavioral metrics that are typically neglected or analyzed separately, and quantifying representations of self and others, which are traditionally evaluated using either self-report or difficult-to-obtain neural measures.

Unlike previous work that showed physical effort parabolically discounts rewards^16^, here cognitive effort discounted rewards linearly, suggesting that cognitive and physical effort may discount rewards in different ways, possibly via dissociable mechanisms^20^, but see Ref.^21^. Consistent with our findings, other work has also found linear models best described cognitive effort discounting^57^. On the other hand, some have found that while physical effort is discounted parabolically, cognitive effort discounts the value of rewards hyperbolically^19^, while others report that both physical and cognitive effort discounting are best described via the power function^15^. Thus, while linear models best described cognitive discounting in our paradigm, determining the functional form of cognitive effort discounting more generally across tasks and contexts requires further research.

Empathy often drives prosocial action^32^ and may have played a role in the current study. While participants did not observe any emotions directly, they may have used perspective taking to imagine the potential happiness or suffering of charities and strangers when first learning for whom they will be earning funds. The extent to which individuals initially took the perspective of charities and strangers may have increased the extent to which their representation of that target overlapped with their own self representation^38,58,59^. When actually deciding to invest effort or not, participants had only 5 s to integrate information about the target, the value of the reward, and the level of effort required. Participants took longer to decide for charities and strangers, but longer choice times were not associated with increased willingness to exert effort for others (Online SM, Figs. S24–S26). Further, longer decision times were associated with increased likelihood of avoiding effort when deciding for the self (Online SM, Figs. S24–S26). In sum longer choice times did not predict effortful choice. Overall, results suggest empathy was unlikely to be at play when participants made decisions for each target. At this stage, the extent of overlap between representations for self and others seemed to play a role.

Although speculative, our results suggest that a way to reduce prosocial apathy may be to highlight the similarity of others to oneself. While the costs and benefits of prosocial effort are often fixed or difficult to change, overlap between self and other representations is malleable. Changing perceptions of self-other overlap could therefore reduce prosocial apathy and increase empathy for others, which could increase well-being for everyone involved^3^. While our findings suggest possible avenues for intervention, further work is needed to evaluate their external validity and generalizability^60,61^. Our results emphasize the potential role of overlapping representations of self and other in explaining why people sometimes are willing to exert effort to help others and provide insights into how to promote prosocial behavior. Future work should test this relationship in a context with increased ecological validity, perhaps using an experience sampling approach^62^.

## Limitations

The scaling of cognitive effort in our task is not anchored to objective units like time in delay discounting^63^ or percent maximal contraction in physical effort^12^. Thus, although we found cognitive effort discounting was linear, it may be parabolic if our design included wider ranges of effort. In other words, it is possible that discounting took a linear rather than a parabolic form not because it was cognitive rather than physical effort per se, but because of the small difference between the units of effort used in the experimental task. However, our results show that self-reported effort varied (on a 1–7 rating scale) substantially from Add 1 (Study 1: M = 1.77, SD = 1.40; Study 2: M = 2, SD = 1.77) to Add 7 (Study 1: M = 6.26, SD = 2.08; Study 2: M = 6.23, SD = 2.27), indicating our findings could not be attributed entirely to insufficient range in cognitive effort.

The multivariate pattern analysis approach we took here to measuring the extent of representational overlap between self and others has several advantages, but also some important limitations. For one, we did not concurrently measure self-other overlap itself with a classic measure like the Inclusion of Other in the Self Scale^24^. However, research suggests the overlapping representations dimension of self-other overlap is itself associated with increased care for the other^38^. Furthermore, similar measures of representational overlap to those we use here have been shown to track with social closeness^27^ and costly prosocial behaviour^39^. Finally, the unobtrusive multivariate approach we take here avoids issues with social desirability that are associated with self-report.

The two experiments described in this study involved samples that were predominantly female and primarily young adult undergraduate students. As such, the generalizability to the general population, and to other non-WEIRD populations^64^, remains to be demonstrated. Males and females differ in both their level and manner of prosocial behaviour, with females often reporting greater concern for others^3^ and more frequent prosocial behaviour^65^ than males. Indeed, supplementary analyses revealed that females in Study 1 were more willing than males to exert effort for their chosen charities (relative to self) (Online SM, Figs. S22, S23), though this effect did not replicate in Study 2. However, since our study was underpowered to detect between-subject effects, more work is needed to understand how males and females differ in their decisions to invest cognitive effort for self and others when multiple non-self targets are involved. In addition, future work should examine whether our findings generalize to older adults, and whether older adults are more willing to invest cognitive effort for others relative to younger adults, as has been observed for physical effort in the lab^17^, but not prosocial behaviour in everyday life^66^.

In this experimental context, we had a high degree of control, and were able to study decisions where both the costs and rewards were real rather than theoretical. On the other hand, this setting may lack ecological validity. In particular, research suggests that individuals may be more likely to help a target if they observe their need directly^32^, and benefit more when they have an opportunity to see the impact of their help^67^. Thus, it remains possible that individuals will exert greater effort for others than the self under some circumstances. Our work suggests that one place this may occur is under conditions with very highly overlapping representations of self and other, such as an individual investing effort for their own child or partner.

## Conclusion

Deciding when to invest effort, and for whom, is an important and recurring problem for any social organism. Despite this importance, decisions about investing effort for others—especially cognitive effort—remain poorly understood. Here we present a paradigm for studying decisions about prosocial cognitive effort. In two preregistered studies we show that, like physical effort, individuals tend to avoid cognitive effort, even at a financial cost. Further, this avoidance is more pronounced when the effort will benefit a charity participants elected to support compared to the self. When working for an intragroup stranger, participants were even less willing to invest mental effort. In sum, while people are miserly with their effort, they are even more miserly when their efforts benefit others, even personally meaningful charities. Individuals varied in their willingness to invest effort for charities and especially strangers. Follow-up multivariate decoding analyses indicated that people who have highly overlapping representations with others may be more willing to act prosocially on their behalf.

## Methods

### Participants

Participants were recruited from the University of Toronto Scarborough’s student participant pool. They earned course credit for participating as well as a bonus Amazon gift voucher (ranging from about $1 to $10 depending on each participant’s choices and performance on the task). Both studies were preregistered and approved by the Research Ethics Review Board. Given prior effect sizes and our repeated-measures design, power analysis suggested a sample of N = 50 would provide 97% statistical power (Westfall, 2016).

In Study 1, we recruited 123 participants. In order to avoid ceiling or floor effects, we pre-registered excluding participants who chose the effortful option more than 85% or less than 15% of all trials (osf.io/rc4an). In total, 71 participants were excluded for selecting the effortful option on 85% or more of trials, and 2 participants were excluded for selecting it on 15% or fewer of trials, leaving us with a sample of N = 51 participants who performed 150 trials each. Our sample was predominantly female (76%) and was composed of young adults (M_age_ = 18.28, SD = 1.27) as is typical of student samples.

In Study 2, we recruited 94 participants. Forty participants were excluded for selecting the effortful choice 85% or more of trials, while 3 participants were excluded for selecting it in 15% or less of trials. In Study 2 five participants were excluded for scoring 30 or less out of 100 on a data quality question, following preregistered criteria (osf.io/ncs97). The result was a sample of N = 47 participants who performed 225 trials each. Our Study 2 sample was also primarily female (76%) and comprised of young adults (M_age_ = 18.40, SD = 1.21).

Given the relatively high exclusion rates in both studies, we reran our mixed-effects modeling, computational modeling, and machine learning analysis with all participants included and found the same overall pattern of effects (see Online SM, Figs. S3, S4, and S11, Tables S4–S7, S10–S12).

### Procedure

All procedures were approved by the University of Toronto Research Ethics board and were subject to relevant ethical guidelines regarding data collection and usage for research with human participants, including the obtainment of informed consent from each participant. All tasks were presented on computer screens using PsychoPy^68,69^. In both studies, participants answered several questionnaires (for full list see Online SM, Table S18), including the Big Five Aspect Scale^52^. Next, participants were told they would be performing a task to earn credits, and that the number of credits they earned would be converted into Canadian dollars and paid out as bonus cash (in the form of Amazon gift vouchers) at the end of the experiment. In Study 1, participants were told they would have an opportunity earn money for themselves and for a charity of their choice. As people are more willing to invest effort for causes that are personally meaningful^70^, participants were given an opportunity to choose which 1 of 5 existing charities (e.g., SickKids Foundation, Canadian Cancer Society) they would like to support. They also had the option to input an alternative charity to the 5 listed if they preferred. Participants were informed that they had to maintain an accuracy level above 90% on the task across all trials to receive the financial compensation for themselves and their selected charity.

We adapted a procedure created by Lockwood and colleagues to study physical effort decisions^16^. Participants made decisions to exert cognitive effort to earn rewards that benefited themselves and others. To quantify the subjective value of cognitive effort for self and other, we systematically varied the amount of cognitive effort required, the reward amount, and whether the reward was given to the participant themselves or charity. In Study 1 we used a 5 (effort levels: 1, 3, 5, 7, 9) × 5 (reward levels: 2, 4, 6, 8, 12) × 2 (target: self, charity) within-subject design.

Prior to testing, participants practiced the task and rated its difficulty. Through this practice and pre-testing procedure, we ensured that our task effort levels actually mapped onto perceived task difficulty. In Study 1 participants rated add 7 to be more effortful (M_Add7_ = 6.26, SD = 2.08; M_Add9_ = 5.02, SD = 3.02; t(245) = 6.52, p < 0.001, r = 0.38) and more frustrating than add 9 (M_Add7_ = 5.80, SD = 2.26; M_Add9_ = 4.85, SD = 3.18; t(245) = 5.07, p < 0.001, r = 0.31). We therefore deviated from our pre-registered plan by coding add 9 as the second most effortful level and add 7 as the most effortful level. Results for the overall model are consistent when tested with 9 coded as the most effortful level (see Online SM, Tables S8 and S9).

In Study 2, we altered our effort levels, asking participants to add 1, 3, 5, 6, or 7 to each of three digits. In addition, we included a third target. Participants made decisions to invest cognitive effort to earn rewards for an intragroup stranger (an unknown other student) intermixed with decisions to invest effort for themselves, and decisions to invest effort for their preferred charity. Participants were told that funds they earned for ‘another student’ would be given to one of their peers in a future study. Thus, in Study 2 we used a 5 (effort levels: 1, 3, 5, 6, 7) × 5 (reward levels: 2, 4, 6, 8, 12) × 3 (target: self, charity, intragroup stranger) within-subject design.

In both studies, participants made 75 decisions for each target (determined pseudo-randomly on each trial; Fig. 1), intermixed in blocks of seventy-five choices. By intermixing decisions for each target within blocks, we control for potential effects of cognitive fatigue on effortful choice over the course of the study^49^. Further, by implementing a within-subject manipulation and using individual participants as a grouping variable, we control for individual differences, such as in arithmetic ability or motivation to engage in mental effort. Participants made 150 decisions in Study 1, and 225 decisions in Study 2. On each trial, participants chose between a static baseline option and a variable alternative option that was more effortful but also more rewarding. The baseline option offered 1 credit for exerting minimal effort (watch and recall a three-digit sequence where all response options were identical and correct). The alternative effortful options required participants to add a number to each digit in a three-digit sequence (e.g., adding 3 to 2, 6, 7 results in 590). More effortful options involved adding larger numbers^22^.

### Analysis

The alpha was set to 0.05 for all statistical tests. We modeled single-trial choice (i.e., baseline and effortful choices coded as 0 and 1, respectively) using a generalized mixed-effects model, with effort, reward, and target and all their two- and three-way interactions as regressors. We fitted the maximal model with participants as a grouping variable^71^, using the glmer function from the lme4 R package^72^: glmer(choice ~ effort × reward × target + (1 + effort × reward × target|participant), family = binomial). Effort and reward variables were normalized to range [− 1, 1] to facilitate effect size comparison. Model statistics were calculated with the summaryh function from the hausekeep package^73^.

To explore time-on-task effects, we added trial number to the model described above and all two-, three-, and four-way interactions. The models were again fitted using the glmer function from the lme4 R package^72^: glmer(choice ~ effort × reward × target × trial + (1 + effort × reward × target × trial|participant), family = binomial).

Maximum likelihood estimation (R function optim) was used to fit linear, parabolic, and hyperbolic effort discounting models^74^. We compared versions of each model with singular and separate *k* discounting parameters, as well as singular and separate *b* softmax parameters for different targets. We compared overall model fit and found consistent results across summed, mean, and median BIC. A linear model with multiple *k* and *b* parameters had the lowest BIC and best described data for the majority of participants in both studies. We ran parameter recovery (see Online SM; Figs. S7–S10) to assess model fit^43–45^.

Linear support vector machine classifiers (SVM; scikit-learn Python library^75^) were fitted separately for each participant and trained to classify whether the target was self or other on any given trial. Models were trained using five stimulus and behavioral features: choice decision time, task accuracy, task reaction time, effort, and reward. We omitted choice as a feature to avoid introducing potential circularities with subsequent analyses where we correlated classification accuracies with willingness to exert effort for others. As robustness checks, we retrained the models with all participants included (ignoring preregistered exclusion criteria), and omitted choice decision time as a feature to eliminate potential circularities; results from these robustness checks were similar (see Online SM; Fig. S11). The SVM classifiers decoded whether a given trial was for self or other, i.e., a charity or stranger. Out-of-sample classification performance (i.e., accuracy) was evaluated using five-fold cross validation. If the multivariate representations of self and other trials overlap substantially, classification accuracies will be around chance level (50%). But when the representations have little overlap in multivariate space, classification accuracies will be greater than chance level. Thus, higher classification accuracies indicate less overlapping representations. To test robustness of this multivariate approach to measuring extent of representational overlap, we also trained SVMs to decode the other features of the stimuli—effort and reward—and examined correlations between self and other representations (see Online SM, Figs. S12, S13).

## Supplementary Information

Supplementary Information.

## Data Availability

Access to materials used and the data-sets generated and analysed during the current study are available in the open science framework repository: https://osf.io/verhb/.
