# Supplementary material for: Cognitive effort for self, strangers, and charities
Source: Sci Rep. 2022 Sep 2;12:15009. doi: 10.1038/s41598-022-19163-y (PMC9440104; doi:10.1038/s41598-022-19163-y)
Supplement: Supplementary file 1 — Supplementary Information. [file 41598_2022_19163_MOESM1_ESM.docx]

**Online Supplementary Material**

*For*

Cognitive effort for self, strangers, and charities

**Additional Analyses**

**Generalized Mixed-Effects Model Results (Maximal Random Effects Structure)**

**Interactions and simple effects.** In Study 1, there was a significant interaction between effort and target, b = 0.28, SE = 0.09, z = 3.00, p = .003, but not reward and target, b = -0.08, SE = 0.09, z = -0.94, p = .347, and no other significant 2 or 3-way interactions (all p’s > .05). Simple effects revealed that effort reduced effortful choice at different rates for self, b = -2.07, SE = 0.07, z = -28.01, p < .001, and charity, b = -1.79, SE = 0.07, z = -26.92, p < .001 (Figure S1A).

In Study 2 we again found a significant interaction between effort and target, b = 0.14, SE = 0.05, z = 2.88, p = .004, but not between reward and target, b = -0.008, SE = 0.05, z = -0.16, p = .871. We examined the simple effects of effort for each target and found that effort reduced effortful choice at different rates for self, b = -1.95, SE = 0.09, z = -21.73, p < .001, charity, b = -1.59, SE = 0.07, z = -24.32, p < .001, and intragroup stranger, b = -1.52, SE = 0.06, z = -23.77, p < .001 (Figure S1B).


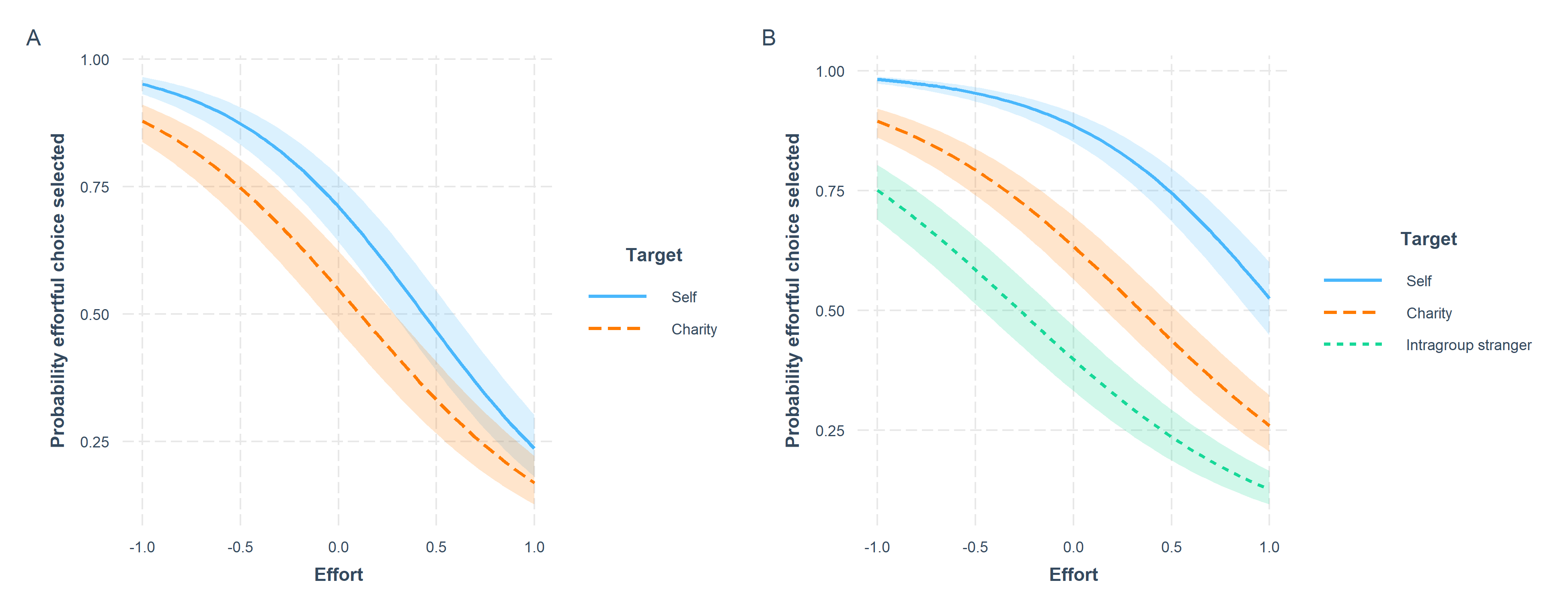


**Figure S1.** Simple effects of effort for each target in Study 1 (A) and Study 2 (B).

**Computational Modeling of Cognitive Effort Discounting**

The behavioral results indicate that cognitive effort discounts potential rewards, but this discounting may vary as a function of the target. To probe this effect, we fitted discounting models using maximum likelihood estimation. We found that a linear discounting model with target-specific *k* discounting and *b* softmax parameters best explained the data. Table S1 shows the mean AIC and BIC values; Table S2 shows the median values; Table S3 shows the summed values.

| **Study** | **Model** | **AIC** | **BIC** |
| --- | --- | --- | --- |
| 1 | Linear - multiple k - multiple b | 110.0136 | 121.8591 |
| 1 | Linear - multiple k - one b | 114.3192 | 123.2033 |
| 1 | Parabolic - multiple k - multiple b | 121.9027 | 133.7482 |
| 1 | Parabolic - multiple k - one b | 128.8416 | 137.7257 |
| 1 | Hyperbolic - multiple k - one b | 148.1198 | 157.0038 |
| 1 | Hyperbolic - multiple k - multiple b | 147.2319 | 159.0774 |
| 2 | Linear - multiple k - multiple b | 178.9027 | 199.1486 |
| 2 | Linear - multiple k - one b | 195.3381 | 208.8354 |
| 2 | Parabolic - multiple k - multiple b | 195.9302 | 216.1761 |
| 2 | Hyperbolic - multiple k - one b | 223.3062 | 236.8035 |
| 2 | Parabolic - multiple k - one b | 225.4997 | 238.997 |
| 2 | Hyperbolic - multiple k - multiple b | 221.2195 | 241.4654 |

**Table S1.** Mean AIC and BIC

| **Study** | **Model** | **AIC** | **BIC** |
| --- | --- | --- | --- |
| 1 | Linear - multiple k - multiple b | 99.04328 | 110.9502 |
| 1 | Linear - multiple k - one b | 107.2879 | 116.0259 |
| 1 | Parabolic - multiple k - multiple b | 115.9831 | 127.972 |
| 1 | Parabolic - multiple k - one b | 120.8243 | 129.6706 |
| 1 | Hyperbolic - multiple k - one b | 145.9771 | 154.9484 |
| 1 | Hyperbolic - multiple k - multiple b | 147.3118 | 159.3007 |
| 2 | Linear - multiple k - multiple b | 178.4239 | 198.9071 |
| 2 | Linear - multiple k - one b | 193.0126 | 206.4514 |
| 2 | Parabolic - multiple k - multiple b | 192.5258 | 213.0224 |
| 2 | Parabolic - multiple k - one b | 224.1969 | 237.5167 |
| 2 | Hyperbolic - multiple k - multiple b | 228.4881 | 248.9848 |
| 2 | Hyperbolic - multiple k - one b | 235.7218 | 249.3862 |

**Table S2.** Median AIC and BIC

| **Study** | **Model** | **AIC** | **BIC** |
| --- | --- | --- | --- |
| 1 | Linear - multiple k - multiple b | 5610.696 | 6214.814 |
| 1 | Linear - multiple k - one b | 5830.279 | 6283.368 |
| 1 | Parabolic - multiple k - multiple b | 6217.039 | 6821.157 |
| 1 | Parabolic - multiple k - one b | 6570.92 | 7024.009 |
| 1 | Hyperbolic - multiple k - one b | 7554.107 | 8007.196 |
| 1 | Hyperbolic - multiple k - multiple b | 7508.829 | 8112.947 |
| 2 | Linear - multiple k - multiple b | 8229.525 | 9160.836 |
| 2 | Linear - multiple k - one b | 8985.553 | 9606.427 |
| 2 | Parabolic - multiple k - multiple b | 9012.79 | 9944.101 |
| 2 | Hyperbolic - multiple k - one b | 10272.09 | 10892.96 |
| 2 | Parabolic - multiple k - one b | 10372.99 | 10993.86 |
| 2 | Hyperbolic - multiple k - multiple b | 10176.1 | 11107.41 |

**Table S3.** Summed AIC and BIC

Wheras physical effort is discounted parabolically (Lockwood et al., 2017), here, cognitive effort discounts rewards linearly. For most participants, linear models provided the best fit in both Study 1 (66.7%) and Study 2 (69.6%; Figure S2). Our winning model is the linear model $subjective\text{ }value=reward*(1-k_{target}\times effort)$, where both the *k* and *b* parameters are different for the separate targets.

**
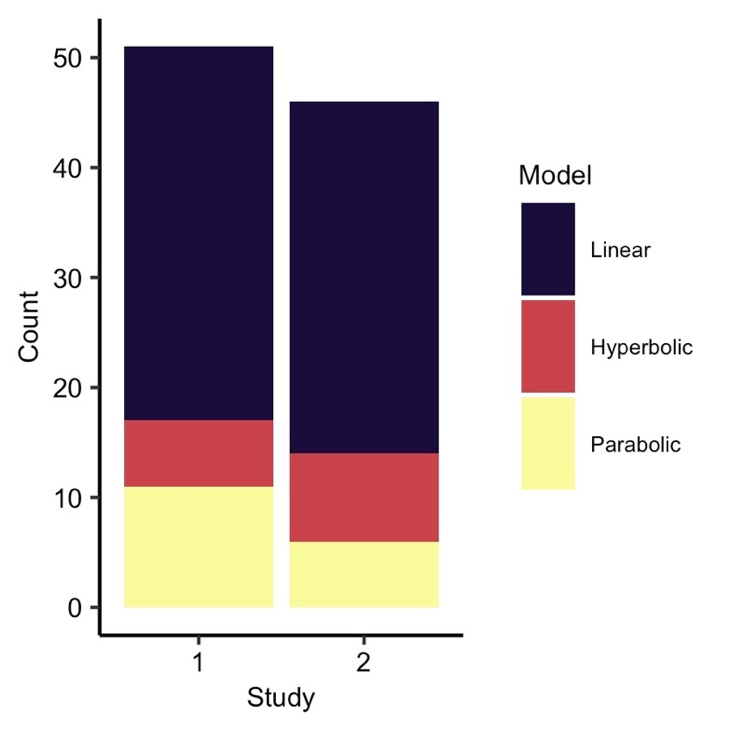
**

**Figure S2.** Count of participants for which each model type provided the best fit.

**Mental Math Task Performance Depended on Effort, Reward, and Target**

**More effortful trials increased reaction times.** In Study 1, participants responded more slowly on effortful compared to baseline (add 0) trials, b = 0.64, SE = 0.04, t(69) = 17.23, p < .001, r = 0.90, and as the amount of effort required increased, b = 0.12, SE = 0.01, t(7253) = 9.74, p < .001, r = 0.11. Likewise, participants responded more slowly on effortful trials, b = 0.49, SE = 0.04, t(87) = 11.42, p < .001, r = 0.77, and as effort increased on effortful trials, b = 0.09, SE = 0.02, t(9900) = 5.59, p < .001, r = 0.06, in Study 2.

**Faster reaction times when working for charity.** We hypothesized that participants would be more engaged when working for themselves and thus have faster reaction times on the mental math task than when working for others. However, contrary to our hypothesis, participants in Study 1 had slightly *slower* reaction times on effortful trials where they earned benefits for themselves (M = 1.08 s, SD = 0.54 s), relative to a charity (M = 1.06 s, SD = 0.53 s; b = -0.04, SE = 0.02, t(4252) = -2.20, p = .028, r = 0.03). In Study 2 participants had comparable reaction times for themselves (M = 1.02 s, SD = 0.53 s), relative to another student (M = 1.01 s, SD = 0.52 s; b = -0.02, SE = 0.02, t(6003) = -1.22, p = .223, r = 0.02), but were again slightly slower for themselves relative to charity (M = 0.98 s, SD = 0.49 s; b = -0.04, SE = 0.02, t(5990) = -2.49, p = .013, r = 0.03).

**Accuracy varied as a function of effort and target.** Participants were less accurate as effort increased [study 1: b = -0.71, SE = 0.11, z = -6.18, p < .001; study 2: b = -0.69, SE = 0.11, z = -6.57, p < .001]. We hypothesized that participants would be less accurate when working for others relative to themselves. Participants were not significantly more accurate on self relative to charity trials [study 1: b = -0.06, SE = 0.11, z = -0.53, p = .597, r = -0.02; study 2: b = -0.17, SE = 0.11, z = -1.57, p = .115, r = -0.05]. However, they were significantly more accurate on self relative to intragroup stranger trials [study 2: b = -0.30, SE = 0.12, z = -2.59, p = .009, r = -0.08].

**Robustness Checks**

**No Exclusions**

Given the high rate of exclusions according to our pre-registered criteria, we ran our analysis with all participants included and obtained a very similar pattern of effects. Shown below are tables from identical models run on included and all participants in both Study 1 and Study 2 (Tables S4-S7). The estimates in the tables below are log-odds and the reference category for the target variable is self.

| **Term** | **Results** |
| --- | --- |
| (Intercept) | b = 0.90, SE = 0.16, z = 5.59, p < .001, r = 0.24 |
| Effort | b = -2.07, SE = 0.07, z = -28.01, p < .001, r = -0.50 |
| Reward | b = 0.51, SE = 0.07, z = 7.86, p < .001, r = 0.14 |
| Target (Charity) | b = -0.71, SE = 0.06, z = -11.27, p < .001, r = -0.19 |
| Effort x Reward | b = -0.07, SE = 0.10, z = -0.67, p = .500, r = -0.02 |
| Effort x Target (Charity) | b = 0.28, SE = 0.09, z = 2.99, p = .003, r = 0.08 |
| Reward x Target | b = -0.08, SE = 0.09, z = -0.95, p = .344, r = -0.02 |
| Effort x Reward x Target (Charity) | b = 0.21, SE = 0.13, z = 1.60, p = .110, r = 0.06 |

**Table S4.** Study 1 results (reported in main text) with preregistered exclusion criteria excepting accuracy. N = 7,300 observations, 51 participants. AIC = 6960.14, Marginal/Conditional R^2^ = 0.32/0.50.

| **Term** | **Results** |
| --- | --- |
| (Intercept) | b = 3.68, SE = 0.31, z = 12.06, p < .001, r = 0.71 |
| Effort | b = -1.94, SE = 0.07, z = -29.73, p < .001, r = -0.47 |
| Reward | b = 0.51, SE = 0.06, z = 8.51, p < .001, r = 0.14 |
| Target (Charity) | b = -0.65, SE = 0.06, z = -11.50, p < .001, r = -0.18 |
| Effort x Reward | b = -0.03, SE = 0.09, z = -0.34, p = .730, r = -0.01 |
| Effort x Target (Charity) | b = 0.34, SE = 0.08, z = 4.13, p < .001, r = 0.09 |
| Reward x Target | b = -0.07, SE = 0.08, z = -0.89, p = .375, r = -0.02 |
| Effort x Reward x Target (Charity) | b = 0.10, SE = 0.11, z = 0.84, p = .401, r = 0.03 |

**Table S5.** Study 1 results with all participants included. N = 17,851 observations, 123 participants. AIC = 9462.52. Marginal/Conditional R^2^ = 0.12/0.79.

| **Term** | **Results** |
| --- | --- |
| (Intercept) | b = 2.05, SE = 0.15, z = 13.38, p < .001, r = 0.49 |
| Effort | b = -1.90, SE = 0.10, z = -19.89, p < .001, r = -0.46 |
| Reward | b = 0.34, SE = 0.10, z = 3.56, p < .001, r = 0.09 |
| Target (Charity) | b = -1.74, SE = 0.08, z = -21.36, p < .001, r = -0.43 |
| Target (Intragroup stranger) | b = -2.60, SE = 0.08, z = -31.58, p < .001, r = -0.58 |
| Effort x Reward | b = -0.009, SE = 0.13, z = -0.07, p = .943, r = 0 |
| Effort x Target (Charity) | b = 0.50, SE = 0.11, z = 4.39, p < .001, r = 0.14 |
| Effort x Target (Intragroup stranger) | b = 0.56, SE = 0.11, z = 4.99, p < .001, r = 0.15 |
| Reward x Target (Charity) | b = -0.12, SE = 0.11, z = -1.09, p = .275, r = -0.03 |
| Reward x Target (Intragroup stranger) | b = -0.13, SE = 0.11, z = -1.15, p = .251, r = -0.04 |
| Effort x Reward x Charity | b = 0.03, SE = 0.16, z = 0.19, p = .849, r = 0.01 |
| Effort x Reward x Intragroup stranger | b = -0.03, SE = 0.16, z = -0.19, p = .848, r = -0.01 |

**Table S6.** Study 2 results (reported in main text) with preregistered exclusion criteria excepting accuracy. N = 10,003 observations, 47 participants. AIC = 9480.36. Marginal/Conditional R2 = 0.37/0.50.

| **Term** | **Results** |
| --- | --- |
| (Intercept) | b = 4.12, SE = 0.34, z = 12.08, p < .001, r = 0.75 |
| Effort | b = -1.92, SE = 0.08, z = -24.22, p < .001, r = -0.47 |
| Reward | b = 0.31, SE = 0.08, z = 4.06, p < .001, r = 0.09 |
| Target (Charity) | b = -1.60, SE = 0.07, z = -23.32, p < .001, r = -0.40 |
| Target (Intragroup stranger) | b = -2.42, SE = 0.07, z = -35.08, p < .001, r = -0.56 |
| Effort x Reward | b = 0.05, SE = 0.11, z = 0.47, p = .636, r = 0.01 |
| Effort x Target (Charity) | b = 0.52, SE = 0.10, z = 5.42, p < .001, r = 0.14 |
| Effort x Target (Intragroup stranger) | b = 0.58, SE = 0.09, z = 6.18, p < .001, r = 0.16 |
| Reward x Target (Charity) | b = -0.05, SE = 0.09, z = -0.55, p = .581, r = -0.01 |
| Reward x Target (Intragroup stranger) | b = -0.06, SE = 0.09, z = -0.69, p = .488, r = -0.02 |
| Effort x Reward x Charity | b = 0.01, SE = 0.13, z = 0.11, p = .913, r = 0 |
| Effort x Reward x Intragroup stranger | b = -0.02, SE = 0.13, z = -0.13, p = .899, r = 0 |

**Table S7.** Study 2 results with all participants included. N = 20,676 observations, 94 participants. AIC = 13,052.75. Marginal/Conditional R2 = 0.15/0.80.

**
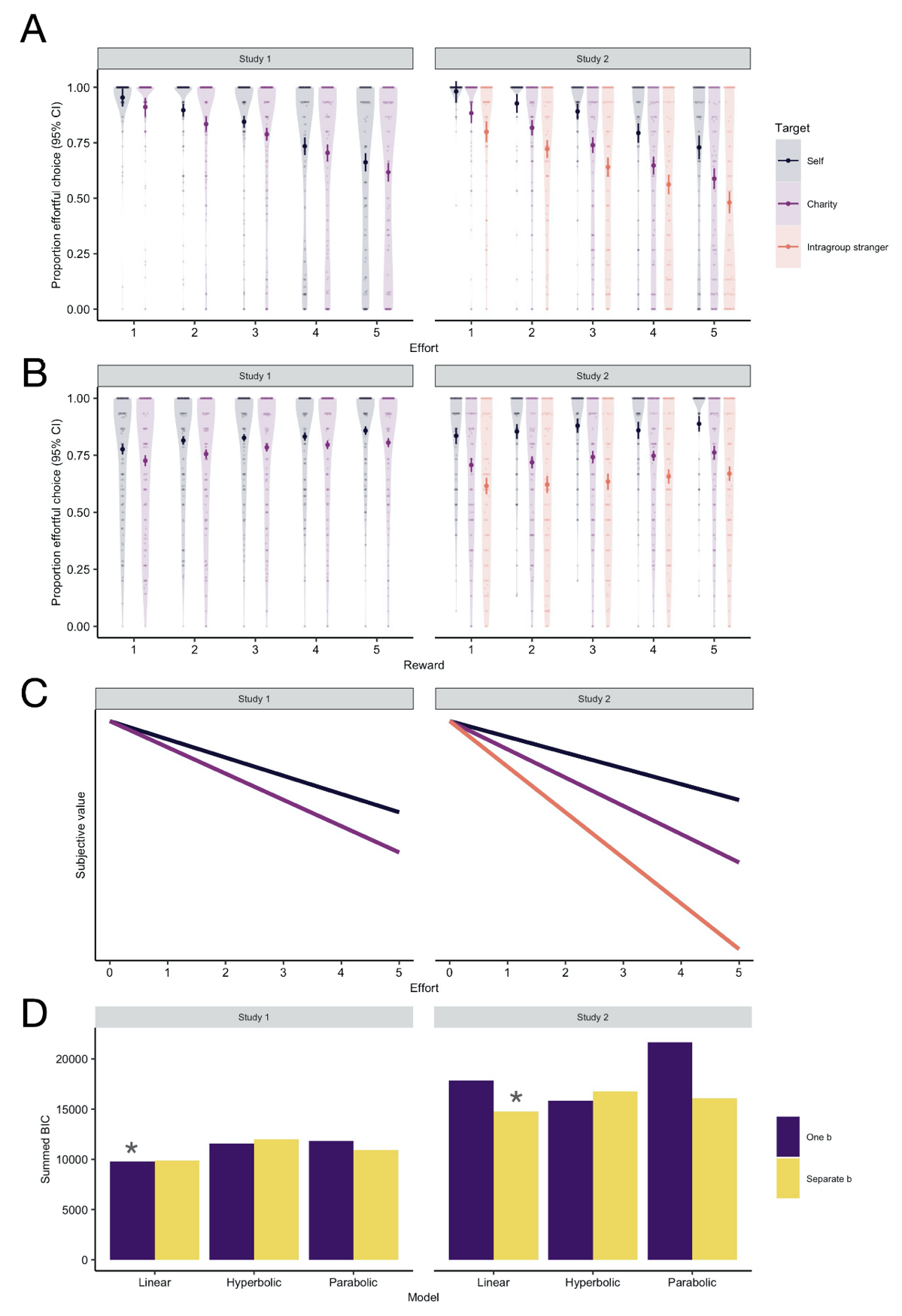
**

**Figure S3.** Proportion of trials where all participants (no exclusions) chose the effortful option (95% CI) rather than the baseline option at different effort levels (A) and reward levels (B) when working for self, charity, and intragroup stranger. Rewards were discounted by cognitive effort by different amounts for self and others. A linear discounting model with separate k discounting and b softmax parameters (C) fitted data best and had the smallest summed BIC values (D).

**Recoding Effort Levels in Study 1**

In Study 1, we re-coded ‘add 7’ as more difficult than ‘add 9’ as this matched the subjective reports of task difficulty participants made after practicing each task. To ensure the robustness of our model to this change, we ran our analysis with both the re-coded (Table S4) and originally coded effort variables (Table S8). The re-coded version shows lower AIC (6960.14) and higher marginal R^2^ (0.32), suggesting better model fit and more explanatory power.

| **Term** | **Results** |
| --- | --- |
| (Intercept) | b = 0.85, SE = 0.15, z = 5.60, p < .001, r = 0.23 |
| Effort | b = -1.78, SE = 0.07, z = -26.07, p < .001, r = -0.44 |
| Reward | b = 0.48, SE = 0.06, z = 7.67, p < .001, r = 0.13 |
| Target (Charity) | b = -0.66, SE = 0.06, z = -10.95, p < .001, r = -0.18 |
| Effort x Reward | b = -0.04, SE = 0.09, z = -0.47, p = .641, r = -0.01 |
| Effort x Target (Charity) | b = 0.27, SE = 0.09, z = 3.04, p = .002, r = 0.07 |
| Reward x Target | b = -0.07, SE = 0.08, z = -0.85, p = .393, r = -0.02 |
| Effort x Reward x Target (Charity) | b = 0.16, SE = 0.13, z = 1.27, p = .204, r = 0.04 |

**Table S8.** Original coded effort study 1. N = 7,298 observations, 51 participants. AIC = 7360.23, Marginal/Conditional R^2^ = 0.27/0.45

**Computational Modeling Results: All Participants**

| **Study** | **Model** | **AIC** | **BIC** |
| --- | --- | --- | --- |
| 1 | Linear – many k – one b | 70.54015 | 79.46780 |
| 1 | Linear – many k – many b | 68.58912 | 80.49265 |
| 1 | Parabolic – many k – many b | 76.81685 | 88.72038 |
| 1 | Hyperbolic – many k – one b | 85.20315 | 94.13080 |
| 1 | Parabolic – many k – one b | 87.20901 | 96.13666 |
| 1 | Hyperbolic – many k – many b | 85.74257 | 97.64610 |
| 2 | Linear – many k – many b | 136.64197 | 156.95219 |
| 2 | Hyperbolic – many k – one b | 155.08808 | 168.62823 |
| 2 | Parabolic – many k – many b | 150.63180 | 170.94202 |
| 2 | Hyperbolic – many k – many b | 157.95878 | 178.26900 |
| 2 | Linear – many k – one b | 176.38420 | 189.92435 |
| 2 | Parabolic – many k – one b | 216.83586 | 230.3760 |

**Table S9.** Mean AIC and BIC for All Participants

| **Study** | **Model** | **AIC** | **BIC** |
| --- | --- | --- | --- |
| 1 | Linear - multiple k - multiple b | 61.17224 | 72.96728 |
| 1 | Linear - multiple k - one b | 67.30640 | 76.33830 |
| 1 | Parabolic - multiple k - multiple b | 80.68016 | 91.96916 |
| 1 | Parabolic - multiple k - one b | 88.40368 | 97.35247 |
| 1 | Hyperbolic - multiple k - one b | 91.90632 | 100.83653 |
| 1 | Hyperbolic - multiple k - multiple b | 92.52323 | 104.43016 |
| 2 | Linear - multiple k - multiple b | 143.69367 | 164.16349 |
| 2 | Parabolic - multiple k - multiple b | 159.86827 | 180.35151 |
| 2 | Hyperbolic - multiple k - one b | 174.00188 | 187.63041 |
| 2 | Linear - multiple k - one b | 184.53638 | 197.62838 |
| 2 | Hyperbolic - multiple k - multiple b | 181.81237 | 202.30897 |
| 2 | Parabolic - multiple k - one b | 236.59336 | 250.12088 |

**Table S10.** Median AIC and BIC for All Participants

| **Study** | **Model** | **AIC** | **BIC** |
| --- | --- | --- | --- |
| 1 | Linear - multiple k - one b | 8676.439 | 9774.540 |
| 1 | Linear - multiple k - multiple b | 8436.461 | 9900.596 |
| 1 | Parabolic - multiple k - multiple b | 9448.472 | 10912.607 |
| 1 | Hyperbolic - multiple k - one b | 10479.988 | 11578.089 |
| 1 | Parabolic - multiple k - one b | 10726.708 | 11824.809 |
| 1 | Hyperbolic - multiple k - multiple b | 10546.336 | 12010.470 |
| 2 | Linear - multiple k - multiple b | 12844.345 | 14753.506 |
| 2 | Hyperbolic - multiple k - one b | 14578.280 | 15851.054 |
| 2 | Parabolic - multiple k - multiple b | 14159.389 | 16068.550 |
| 2 | Hyperbolic - multiple k - multiple b | 14848.126 | 16757.286 |
| 2 | Linear - multiple k - one b | 16580.115 | 17852.889 |
| 2 | Parabolic - multiple k - one b | 20382.571 | 21655.344 |

**Table S11.** Summed AIC and BIC for All Participants


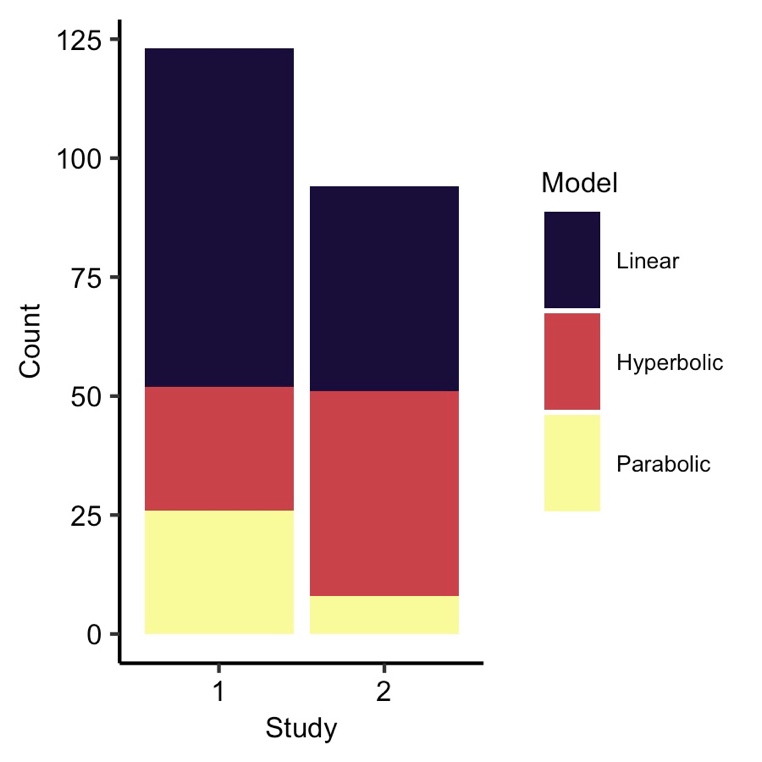


**Figure S4.** Count of all participants (no exclusions) for which each model type provided the best fit.

With all participants included, we again found k discounting parameters were larger for charity than the self (Study 1: b = 0.04, SE = 0.02, t(122) = 2.12, p = .036, r = 0.19; Study 2: b = 0.06, SE = 0.03, t(186) = 2.21, p = .028, r = 0.16). In Study 2, we observed larger k discounting parameters for intragroup stranger relative to the self, b = 0.13, SE = 0.03, t(186) = 5.28, p < .001, r = 0.36, and to charity, b = 0.08, SE = 0.03, t(186) = 3.07, p = .003, r = 0.22 (Figure S5).


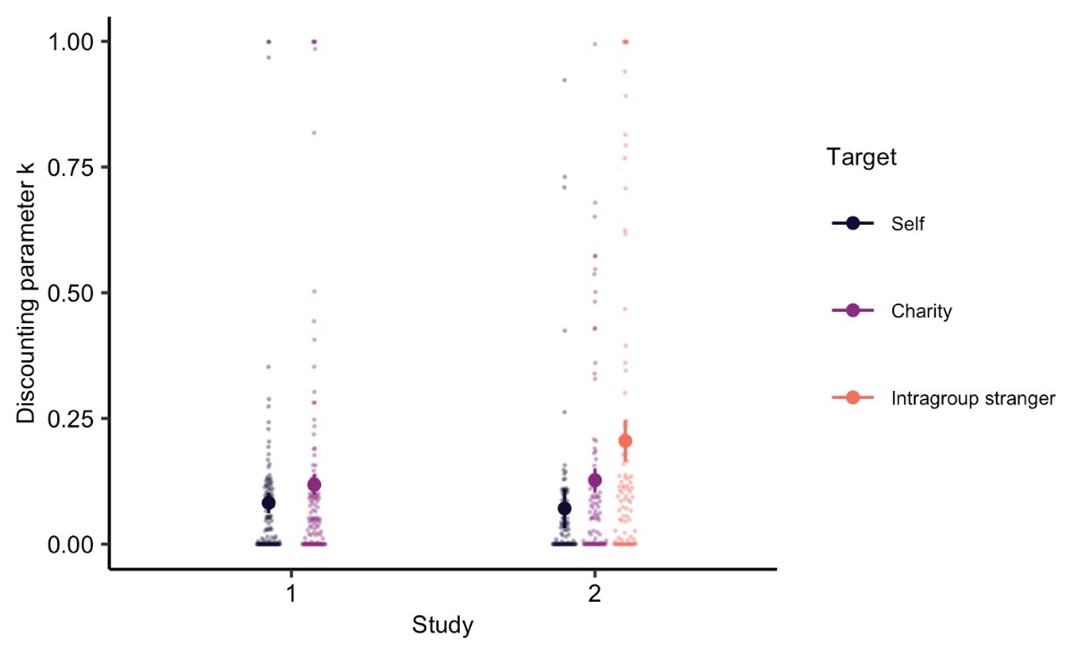


**Figure S5.** Discounting parameters for all participants (no exclusions). Discounting parameters (k) were smallest for self in both Study 1 and Study 2. Each dot is a k value for one participant. Circles represent means and error bars are 95% CI around the mean.

Consistent with prior results, we found b softmax parameters were not significantly different across self and charity trials in Study 1, b = −0.03, SE = 0.08, t(122) = −0.32, p = .753, r = 0.03, but were larger for self than charity, b = −0.26, SE = 0.07, t(186) = −3.48, p < .001, r = 0.25, and intragroup stranger, b = −0.47, SE = 0.07, t(186) = −6.27, p < .001, r = 0.42, in Study 2. Further, b values were higher for charity than strangers, b = −0.21, SE = 0.07, t(186) = −2.79, p = .006, r = 0.20 (Figure S6).


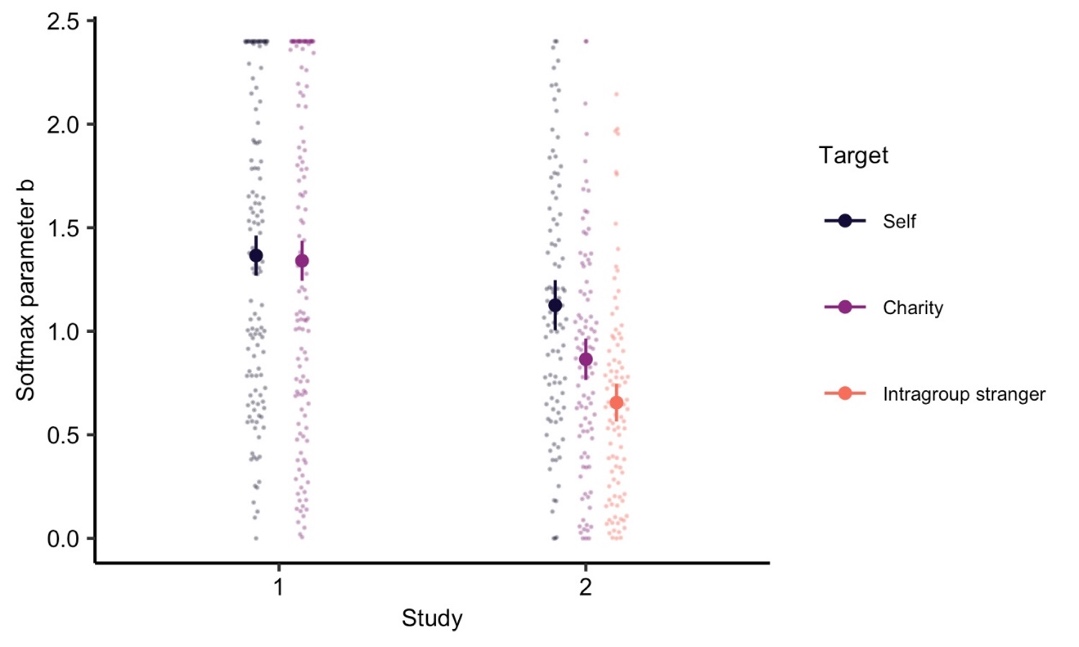


**Figure S6.** Softmax parameters with all participants (no exclusions). Softmax parameters did not differ in Study 1 but were largest for self relative to charity and intragroup stranger in Study 2. Each dot is a b value for one participant. Circles represent means and error bars are 95% CI around the mean.

**Computational Models: Model Fit and Parameter Recovery**

To ensure our model was not only a good fit of the data but a good predictor of behaviour, we performed parameter recovery to check model fit. For each participant, we simulated data using the fitted parameters (100 trials per unique effort-reward pair for each target). We then fitted the model using the maximum likelihood approach described in the main analysis to recover the parameters. The results are shown in the two figures below. With included participants, there is agreement between the fitted and simulated parameter values (Figure S7; Table S12). Crucially, the model-predicted choices also correlated highly with observed choices (Figure S8; Table S13). When analysis is run with no exclusions, we again see agreement between the fitted and simulated parameter values (Figure S8; Table S14), and that model-predicted choices correlate highly with observed choices (Figure S9; Table S15).

**
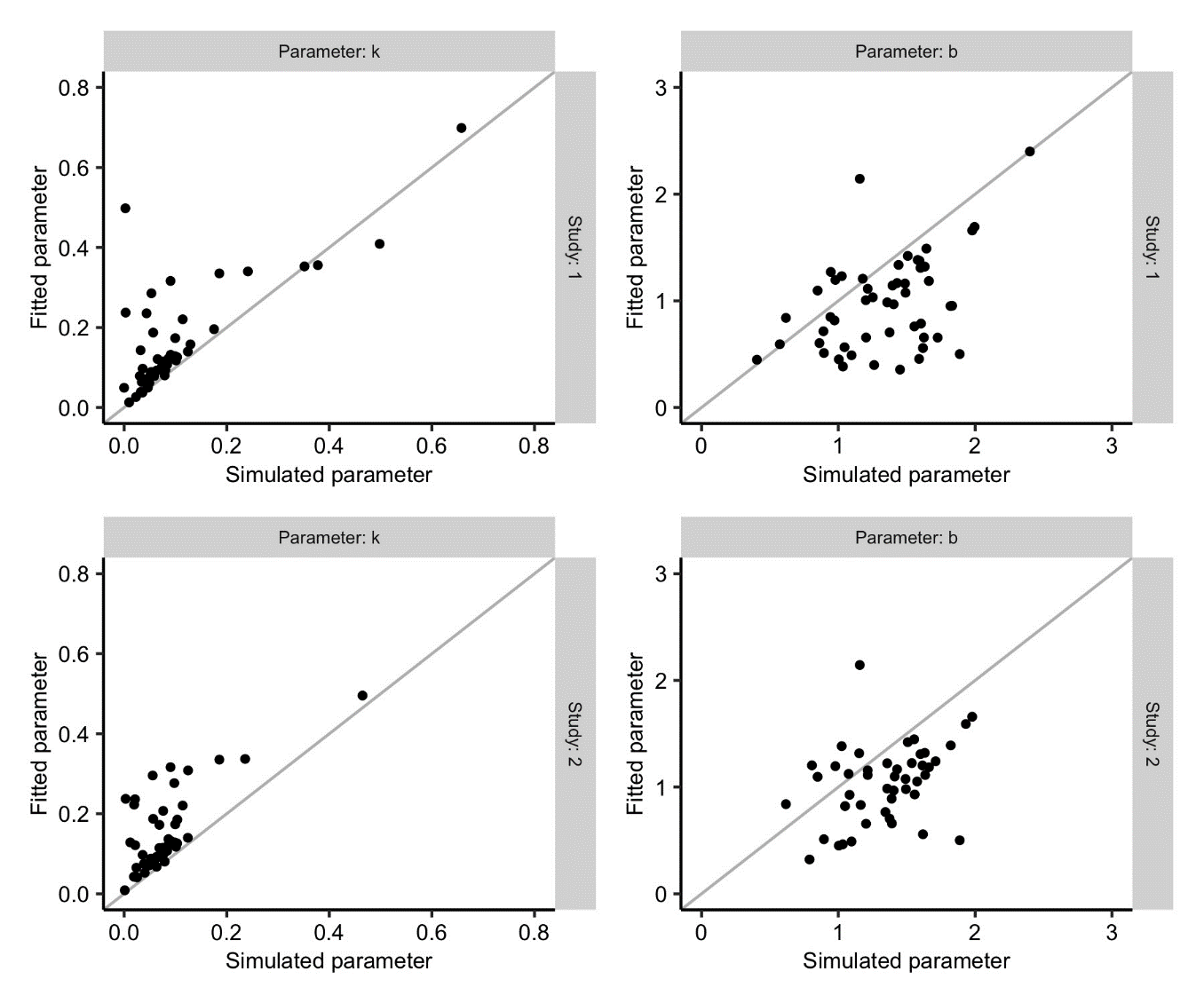
**

**Figure S7.** Parameter recovery for included participants for the linear discounting model with separate k and b parameters for each target. Each dot is one participant. Dots that fall on the diagonal indicate excellent model fit: The simulated parameter values are identical to the fitted parameter values.

| **Study** | **Parameter Term** | **Results** |
| --- | --- | --- |
| 1 | b | b = 0.49, SE = 0.14, t(49) = 3.44, p = .001, r = 0.44 |
| 2 | b | b = 0.39, SE = 0.16, t(44) = 2.47, p = .018, r = 0.35 |
| 1 | k | b = 0.82, SE = 0.10, t(49) = 8.18, p < .001, r = 0.76 |
| 2 | k | b = 0.94, SE = 0.14, t(44) = 6.54, p < .001, r = 0.70 |

**Table S12.** Simulated parameters for included participants correlated with fitted parameters.


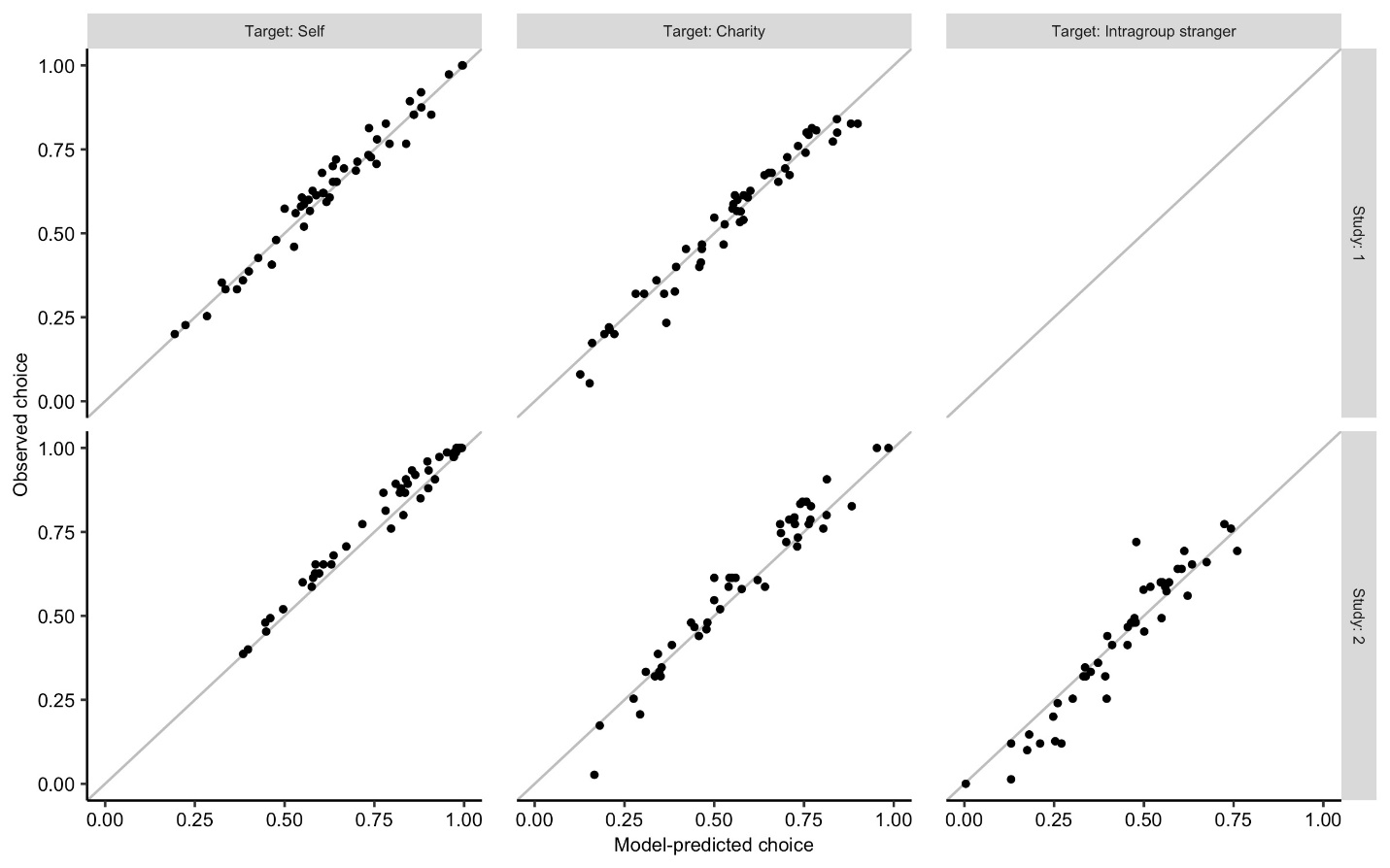


**Figure S8.** Model fit for included participants for the linear discounting model with separate k and b parameters for each target. Each dot is one participant. Dots that fall on the diagonal indicate excellent model fit: The model-predicted choices are identical to the observed choices.

| **Study** | **Target** | **Results** |
| --- | --- | --- |
| 1 | Charity | b = 1.02, SE = 0.03, t(49) = 36.25, p < .001, r = 0.98 |
| 1 | Self | b = 1.00, SE = 0.03, t(49) = 37.12, p < .001, r = 0.98 |
| 2 | Charity | b = 1.09, SE = 0.04, t(44) = 30.55, p < .001, r = 0.98 |
| 2 | Intragroup Stranger | b = 1.16, SE = 0.05, t(44) = 21.76, p < .001, r = 0.96 |
| 2 | Self | b = 0.98, SE = 0.02, t(44) = 41.81, p < .001, r = 0.99 |

**Table S13.** Predicted choices for included participants correlated highly with observed choices for different targets in Study 1 and 2.

**Computational Models: Model Fit and Parameter Recovery for All Participants (No Exclusions)**

**
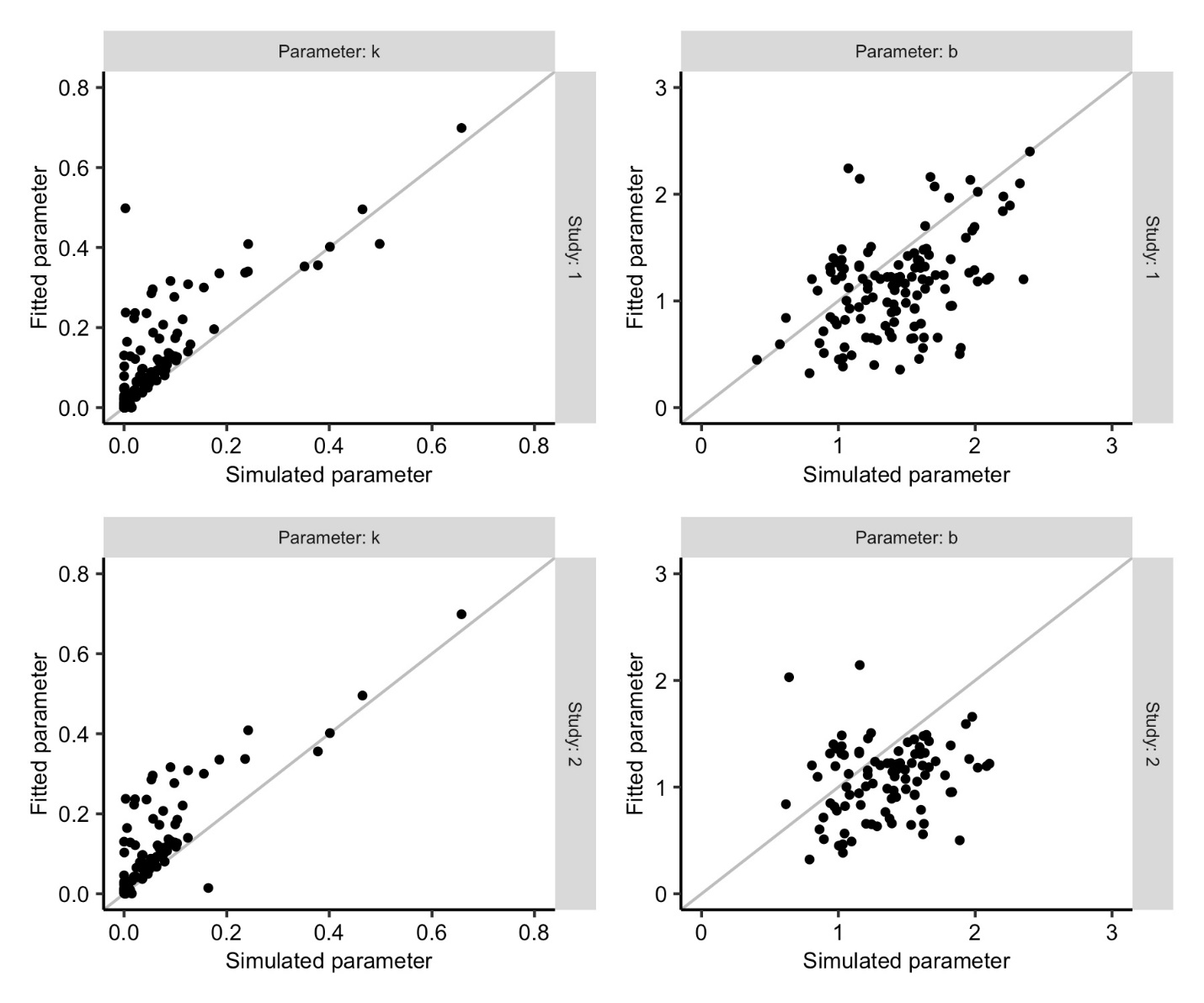
**

**Figure S9.** Parameter recovery for all participants (no exclusions) for the linear discounting model with separate k and b parameters for each target. Each dot is one participant. Dots that fall on the diagonal indicate excellent model fit: The simulated parameter values are identical to the fitted parameter values.

| **Study** | **Parameter Term** | **Results** |
| --- | --- | --- |
| 1 | b | b = 0.51, SE = 0.09, t(121) = 5.58, p < .001, r = 0.45 |
| 2 | b | b = 0.20, SE = 0.10, t(92) = 1.92, p = .058, r = 0.20 |
| 1 | k | b = 0.98, SE = 0.06, t(121) = 15.31, p < .001, r = 0.81 |
| 2 | k | b = 1.01, SE = 0.07, t(92) = 14.30, p < .001, r = 0.83 |

**Table S14.** Simulated parameters for all participants (no exclusions) predicted fitted parameters.


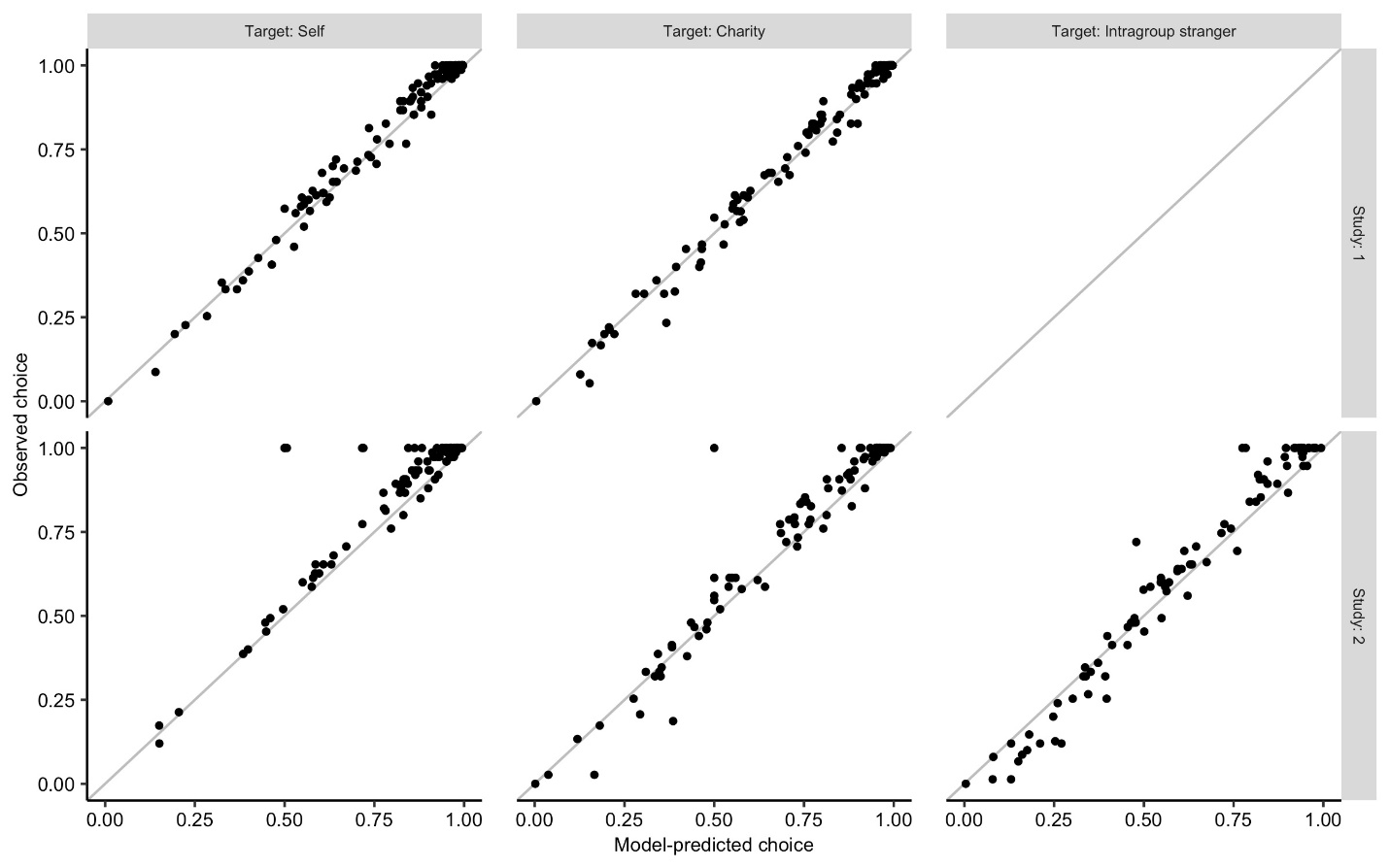


**Figure S10.** Model fit for all participants (no exclusions) for the linear discounting model with separate k and b parameters for each target. Each dot is one participant. Dots that fall on the diagonal indicate excellent model fit: The model-predicted choices are identical to the observed choices.

| **Study** | **Target** | **Results** |
| --- | --- | --- |
| 1 | Charity | b = 1.02, SE = 0.03, t(49) = 36.25, p < .001, r = 0.98 |
| 1 | Self | b = 1.00, SE = 0.03, t(49) = 37.12, p < .001, r = 0.98 |
| 2 | Charity | b = 1.09, SE = 0.04, t(44) = 30.55, p < .001, r = 0.98 |
| 2 | Intragroup Stranger | b = 1.16, SE = 0.05, t(44) = 21.76, p < .001, r = 0.96 |
| 2 | Self | b = 1.13, SE = 0.02, t(92) = 53.52, p < .001, r = 0.98 |

**Table S15.** Predicted choices for all participants (no exclusions) correlated highly with observed choices for different targets in Study 1 and 2.

**Machine Learning Decoding Target: All Participants or Without Choice Decision Time**

As robustness checks, we also refitted the machine learning results reported in the main text with all participants included (i.e., ignoring preregistered exclusions), and without choice decision time as a training feature. We found results consistent with those reported in the main text. With no exclusions, classification accuracies were lower when decoding charity (vs. self) than when decoding stranger (vs. self), b = 0.04, SE = 0.008, t(93) = 4.23, p < .001, r = 0.40, indicating greater self-charity than self-stranger representational overlap. Decoding accuracy was negatively correlated with prosocial effort for charity, b = −1.26, SE = 0.20, t(92) = −6.25, p < .001, r = −0.55, and stranger, b = −1.53, SE = 0.18, t(92) = −8.36, p < .001, r = −0.66. See Figure S11.


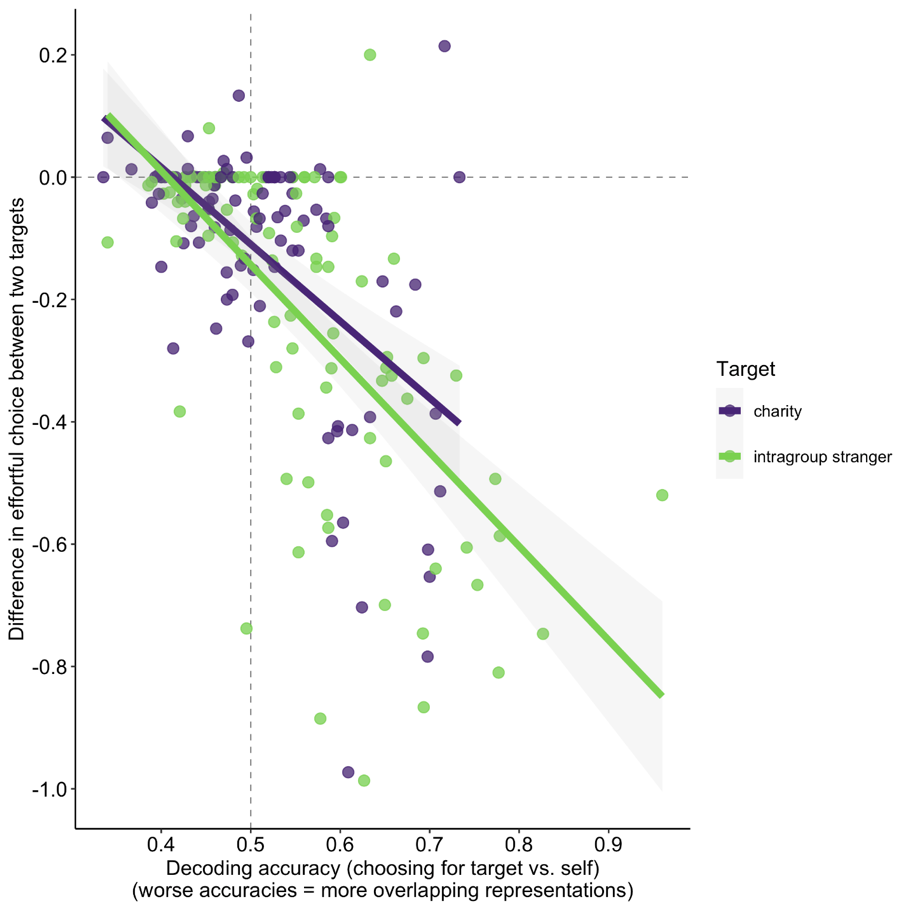


**Figure S11.** Difference in effortful choice between self and other trials as a function of linear support vector machine decoding or classification accuracy (all participants; no exclusions). Dots are individual participants’ data for charity (purple) and stranger (green) trials. Dots below or above the dashed horizontal line are participants who chose the effortful option less or more, respectively, for the other target relative to themselves. The vertical dashed line indicates chance-level classification accuracy (50%) when decoding whether the target on any given trial was self or other target (charity or stranger). Higher classification accuracies indicate less representational overlap and are associated with choosing the effortful option less for others.

Similarly, we found consistent results when choice decision time was not a training feature, such that classifiers were trained only on features relevant to the math task. Decoding accuracy was significantly worse decoding self from charity than from stranger, b = 0.04, SE = 0.01, t(45) = 3.10, p = .003, r = 0.42, indicating increased self-charity representational overlap. Once again, classification accuracy when decoding self from other trials—which indicates reduced overlap of representations—was negatively associated with prosocial effort for both charity, b = −1.40, SE = 0.33, t(44) = −4.20, p < .001, r = −0.54, and stranger, b = −1.73, SE = 0.37, t(44) = −4.70, p < .001, r = −0.58. Results suggest consistently significant and large effects, illustrating the robustness of our exploratory machine learning analysis.

**Quantifying Representational Overlap by Decoding Effort and Reward**

To further explore the robustness of our results linking overlap of representational overlap and individual willingness to invest prosocial effort, we trained two additional sets of machine learning models (linear support vector machines) on multivariate behavioural data to classify two other components of the stimulus that was shown on each trial: effort and reward levels. We decoded pairs of effort (or reward) levels (see Fig. S12A) and represented the decoding/classification accuracies in matrices. Out-of-sample classification performance (i.e., accuracy) was evaluated using five-fold cross validation and correlations between decoding matrices were computed using the Mantel test (Mantel, 1967), and converted from correlation r to Fisher’s z’ before performing analyses. The correlations between these decoding matrix representations on self and other trials were used as an index of extent of overlapping representations. The cross-validated classification accuracies are represented in matrices (Fig. S12A, 12B), which can be interpreted as participants' representations of different targets (e.g., Popal et al., 2019). We first trained classifiers on 5 features: choice, choice decision time, task reaction time, task accuracy, and effort/reward. Classifiers showed good performance decoding effort (Figure S12A), but not reward (Figure S12B). Interestingly, the extent to which classifiers trained on self trials and classifiers trained on other trials overlapped was a significant predictor of prosocial effort for the overlapping other (Figure S12C). When trained on 5 features, representational overlap was associated with increased prosocial effort for intragroup strangers, b = 0.27, SE = 0.05, t(43) = 5.72, p < .001, r = 0.66, and charity, b = 0.10, SE = 0.05, t(43) = 2.02, p = .050, r = 0.29.


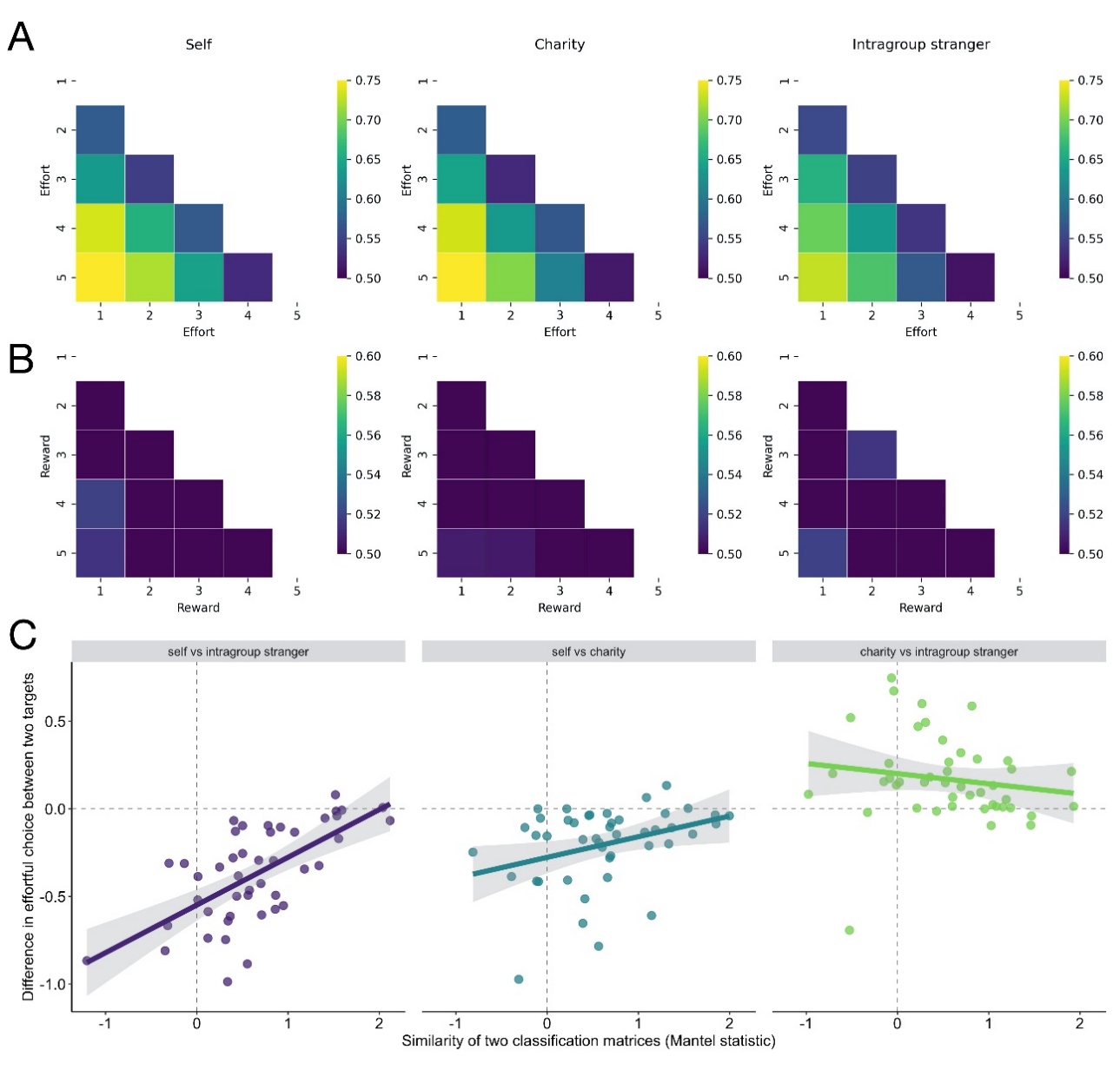


**Figure S12.** Classification matrices representing cross-validated accuracies of support vector machines trained on 5 features (effort/reward, choice, choice decision time, task reaction time, task accuracy) for different pairs of effort levels (A) and reward levels (B). The models correctly classified the most different pairs of effort levels (1 vs. 5) about 75% of the time, but similar pairs (4 vs. 5) only slightly above chance level (50%). Brighter cells indicate better cross-validated pairwise classification accuracies. Similarity of charity or intragroup stranger matrices to self matrix (C) correlated with willingness to exert effort for charity or intragroup stranger. Each dot is one participant.

We also trained classifiers without choice as a training feature (to avoid potential circularity in the analysis) to classify pairs of effort and reward levels (features: effort/reward, decision time, task accuracy, task reaction time). The classifiers accurately decoded different effort levels (Fig. S13A) but not reward levels (Fig. S13B) above chance accuracy (0.50), consistent with the finding in main text that reward influenced choices less than effort.

We again found self-other representational overlap with a stranger predicts prosocial effort on their behalf. There was a significant interaction between overlap and target, F(2, 129) = 3.67, p = .028, in that participants with greater self-stranger overlap chose the effortful option more frequently for the stranger (r = .37, p = .012; Fig. S13C first panel). In fact, participants with the greatest overlap of self-other representations chose the effortful option for the stranger nearly as often, but not more often, as for themselves.

Both overlap of self-other representations and prosocial effort were higher in charity trials relative to intragroup stranger trials, which is unsurprising as participants chose a personally meaningful charity to support. However, the extent of self-charity overlap was not associated with prosocial effort for charity (r = -0.02, p = .91; Fig S13C second panel). This may indicate that the relationship between representational overlap and prosocial effort is only observed when the difference in effortful choice between self and other is large, and self-other representational overlap is highly variable. Further, when the self-representation was irrelevant—when computing similarity between intragroup stranger and charity matrices (Fig. S13C third panel)—matrix similarity did not correlate with choice (r = .16, p = .309), even with choice as a training feature, suggesting the process may be inherently self-referential (Meyer & Lieberman, 2018; Tamir & Mitchell, 2010).


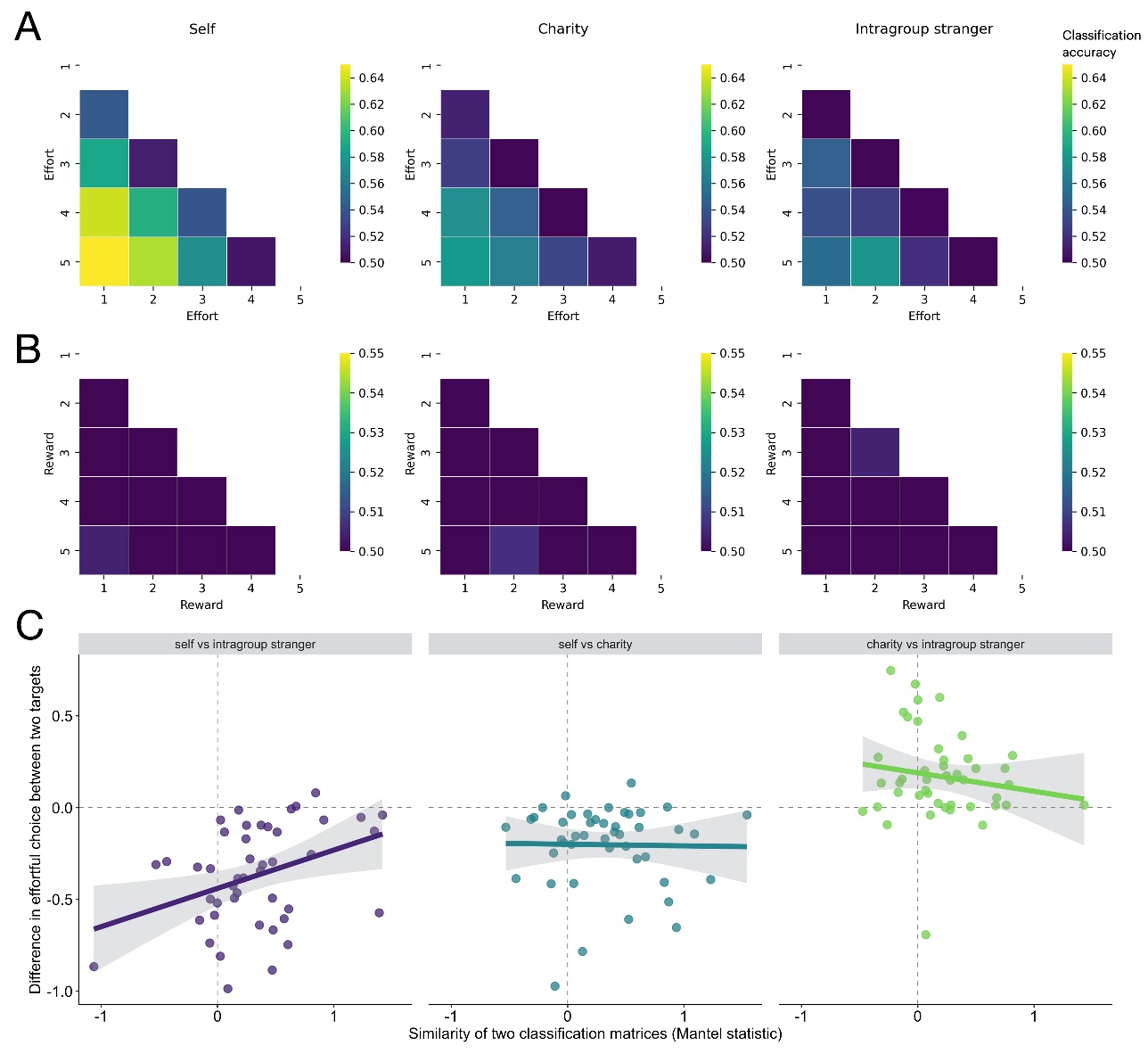


**Fig. S13.** Matrices representing cross-validated classification accuracies for different pairs of effort levels (A) and reward levels (B) for models trained without choice. The models correctly classified the most different pairs of effort levels (1 vs. 5) about 64% of the time, but similar pairs (4 vs. 5) were closer to chance level (0.50). Brighter cells indicate better cross-validated pairwise classification accuracies. Similarity of matrices for others (either stranger or charity) to self-matrix (C) correlated with willingness to exert effort, but only for intragroup strangers and not for charities. Each dot is one participant.

**Exploring Feature Weights**

We obtained feature weights from the models to determine which features were driving discrimination of effort (Figure S13) and reward (Figure S14) pairs. The models could not decode reward pairs above chance even with choice as a training feature (Fig. S14B), the inclusion of choice may introduce circularity into the interpretation. Examining feature weights, we found choice was the primary feature, but not the only important feature driving discrimination.


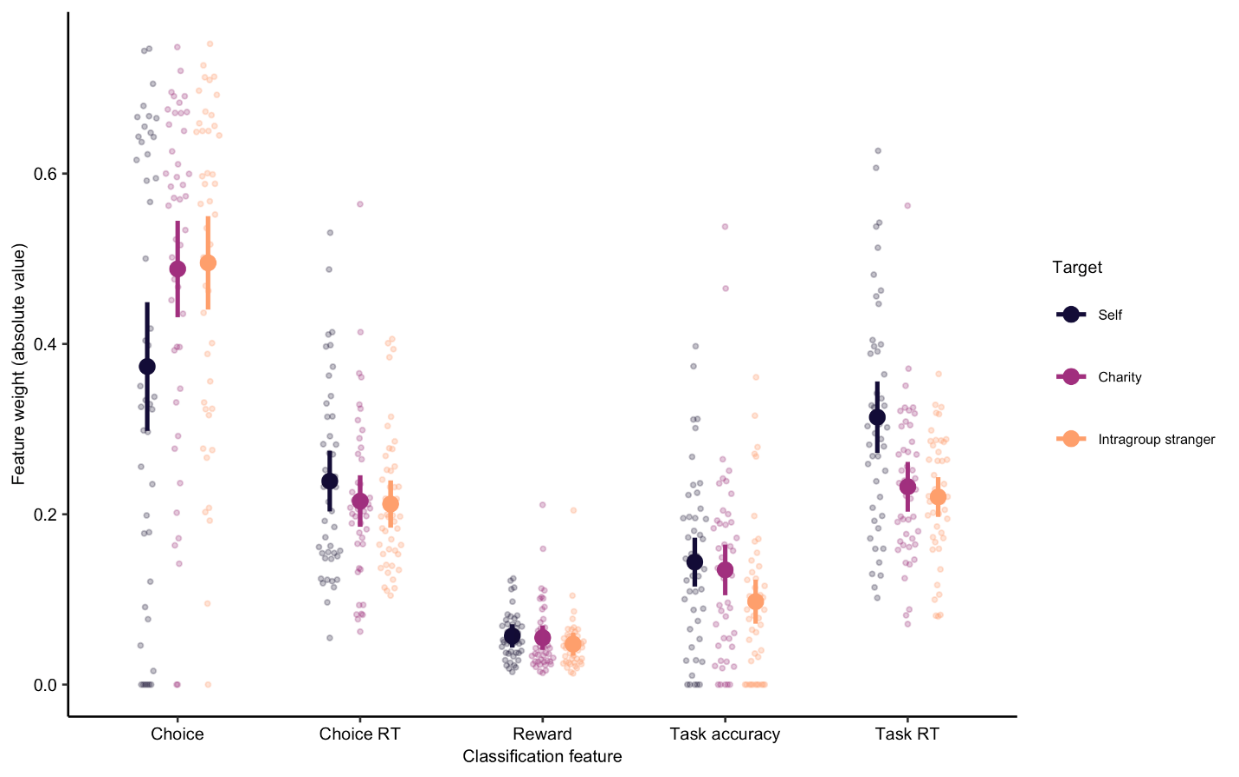


**Figure S14.** Support vector machine feature weights when decoding effort pairs. Choice was included as a feature. Each dot is one participant. Mean and 95% CI error bars are shown.


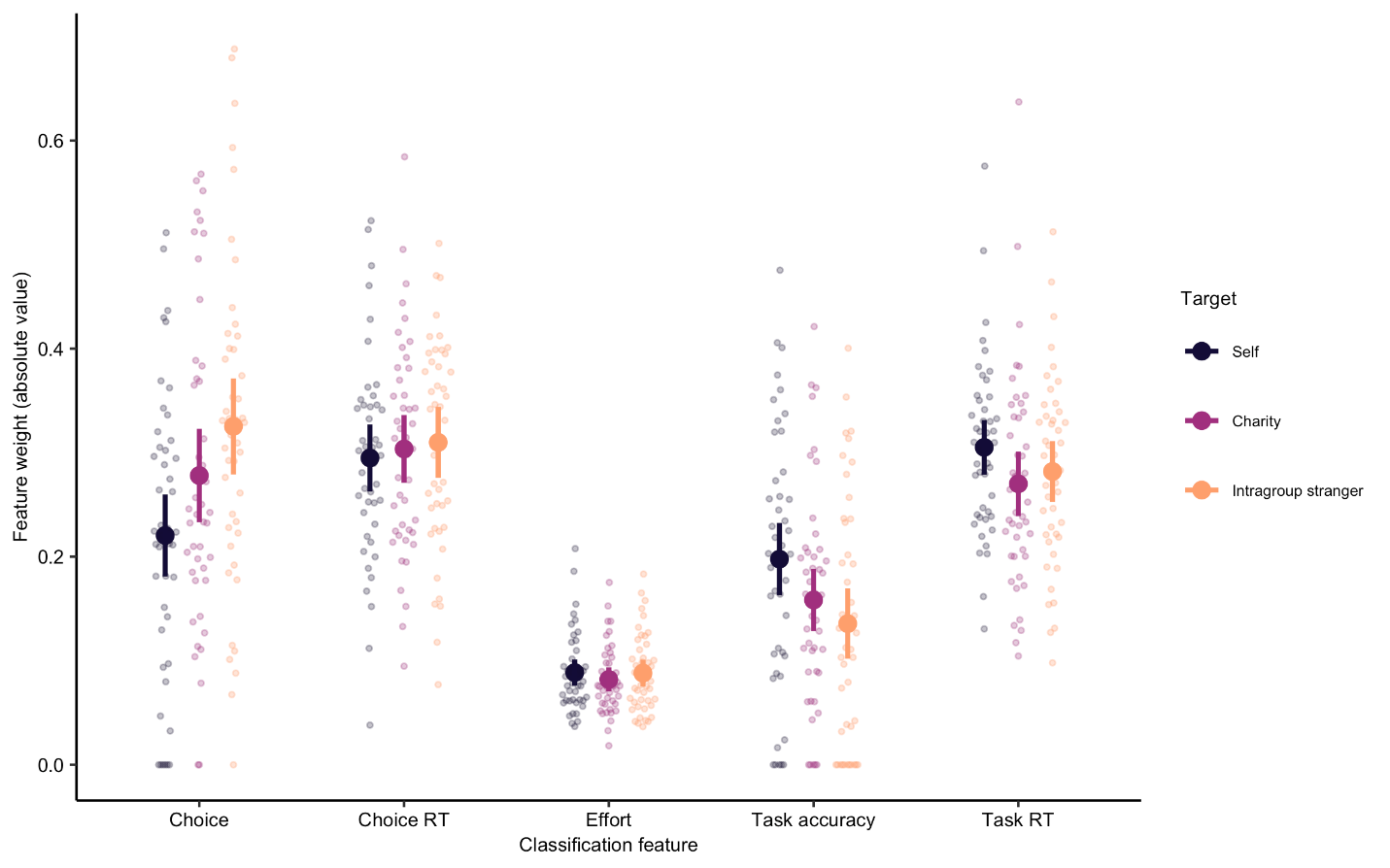


**Figure S15.** Support vector machine feature weights when decoding reward pairs. Choice was included as a feature. Each dot is one participant. Mean and 95% CI error bars are shown.


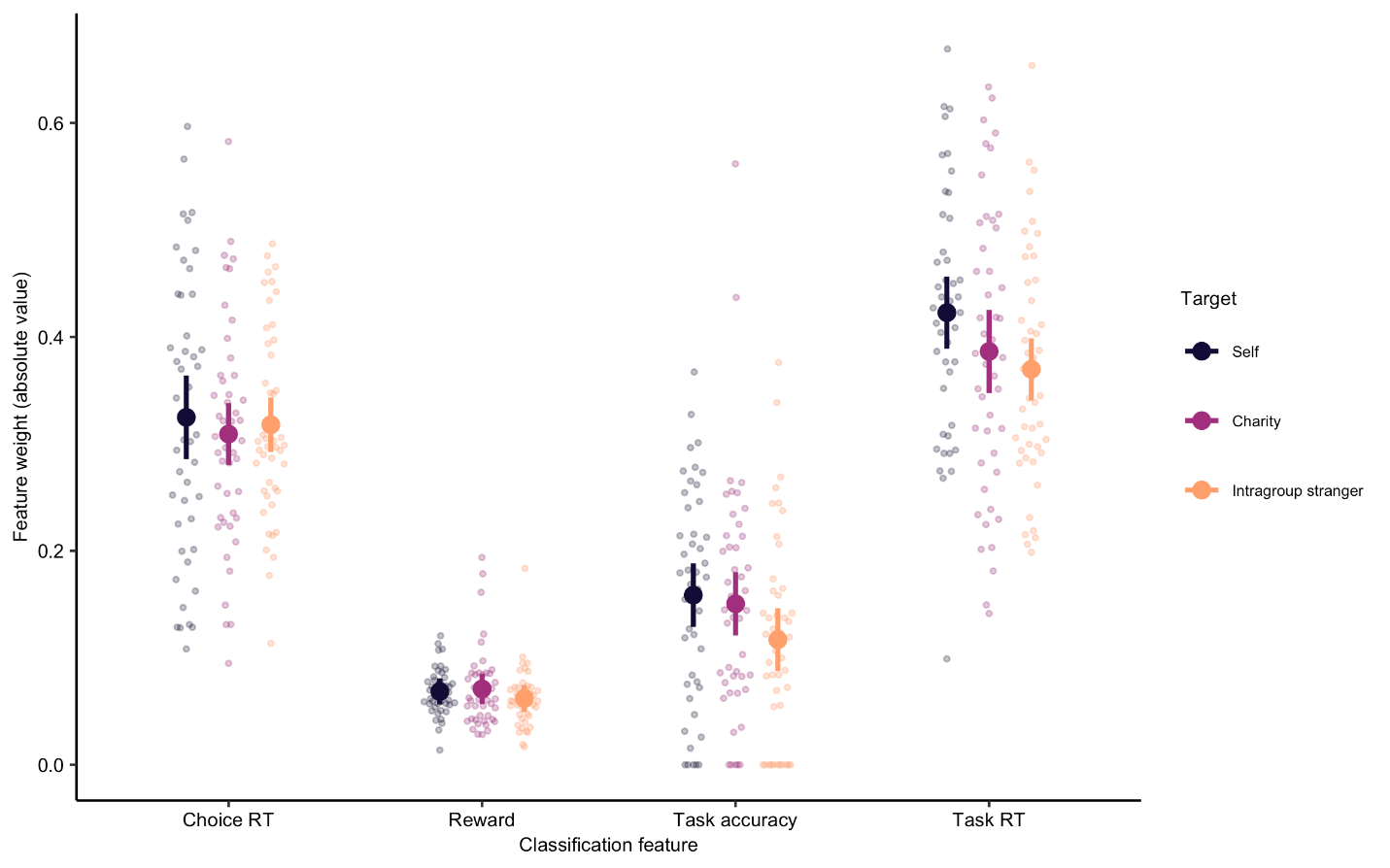


**Figure S16.** Support vector machine feature weights when decoding effort pairs. Choice was excluded as a feature. Each dot is one participant. Mean and 95% CI error bars are shown.


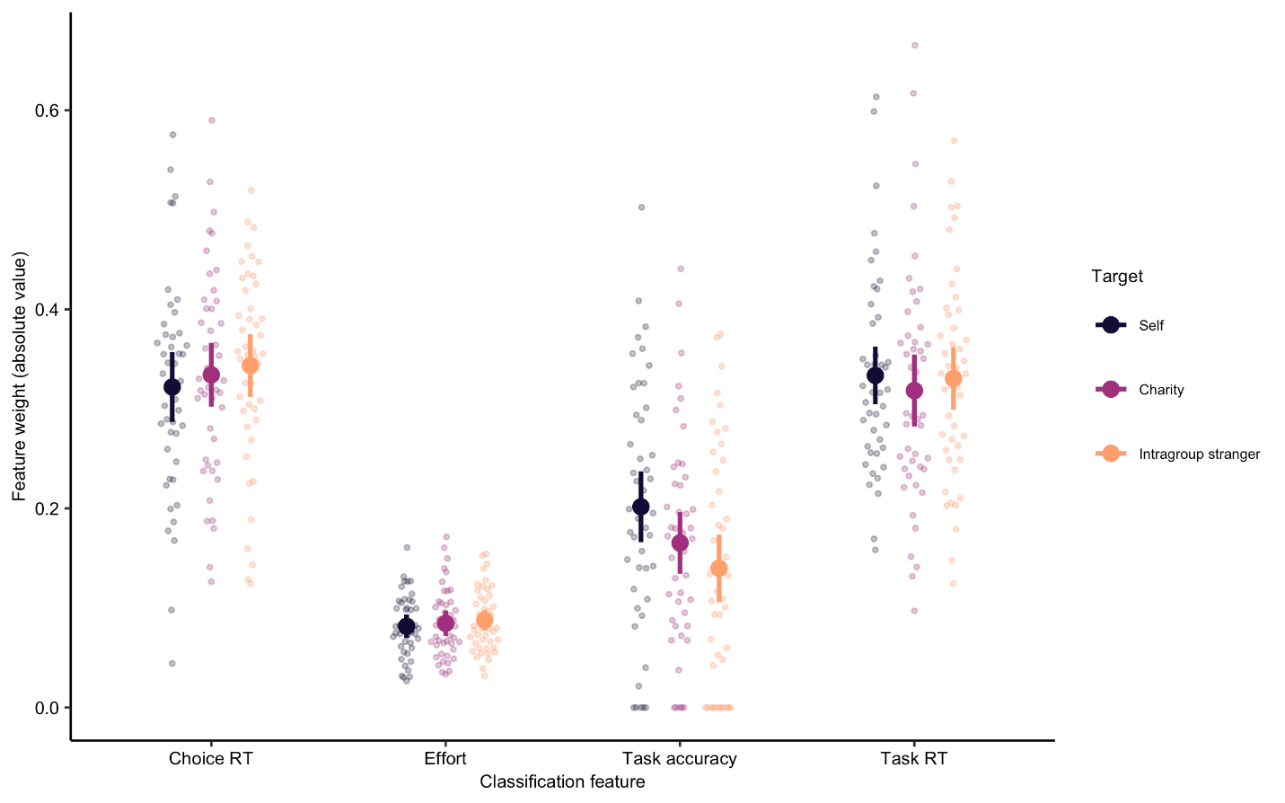


**Figure S17.** Support vector machine feature weights when decoding reward pairs. Choice was excluded as a feature. Each dot is one participant. Mean and 95% CI error bars are shown.

**Decoding Target and Big-5 Compassion**

We tested whether accuracy at decoding self from other trials would be associated with the compassion aspect of the agreeableness factor from the Big 5 (DeYoung et al., 2007). As reported in the main text, we found this relationship was significant for both charity and strangers (Figure S18). When the analysis was run with all participants included, results were robust for charity, b = −0.05, SE = 0.01, t(92) = −3.55, p < .001, r = −0.35, and stranger, b = −0.04, SE = 0.02, t(92) = −2.19, p = .031, r = −0.22; Figure S19. When choice reaction time was not included as a training feature (choice was not included in any models decoding target), the relationship remained significant for charity, b = −0.05, SE = 0.02, t(44) = −2.64, p = .011, r = −0.37, but not stranger, b = −0.03, SE = 0.02, t(44) = −1.35, p = .183, r = −0.20.


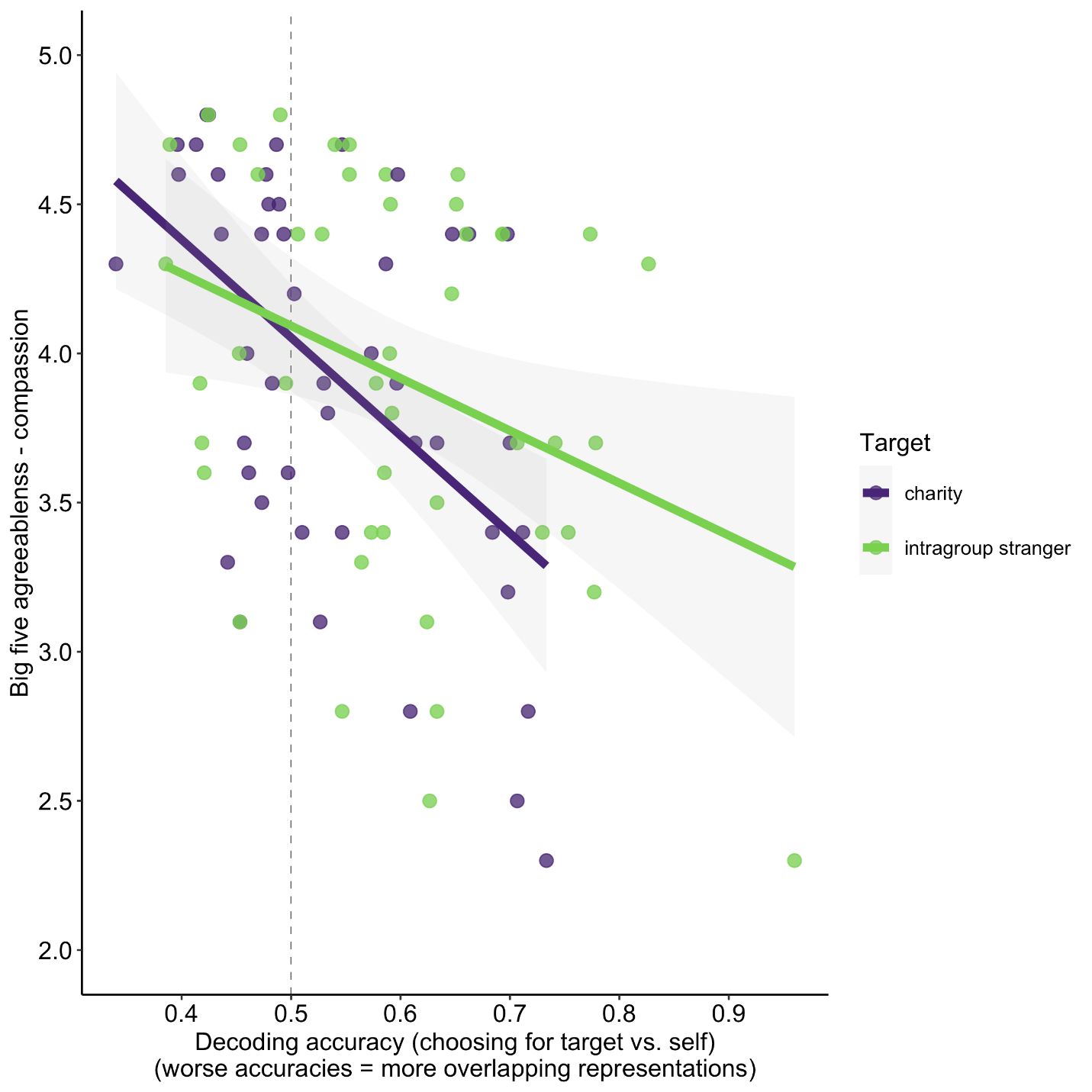


**Figure S18.** Compassion (BFAS) as a function of linear support vector machine decoding or classification accuracy for included participants. Dots are individual participants’ data for charity (purple) and stranger (green) trials. The vertical dashed line indicates chance-level classification accuracy (50%) when decoding whether the target on any given trial was self or other target (charity or stranger). Higher classification accuracies indicate less representational overlap and are associated with reduced compassion at the trait level.


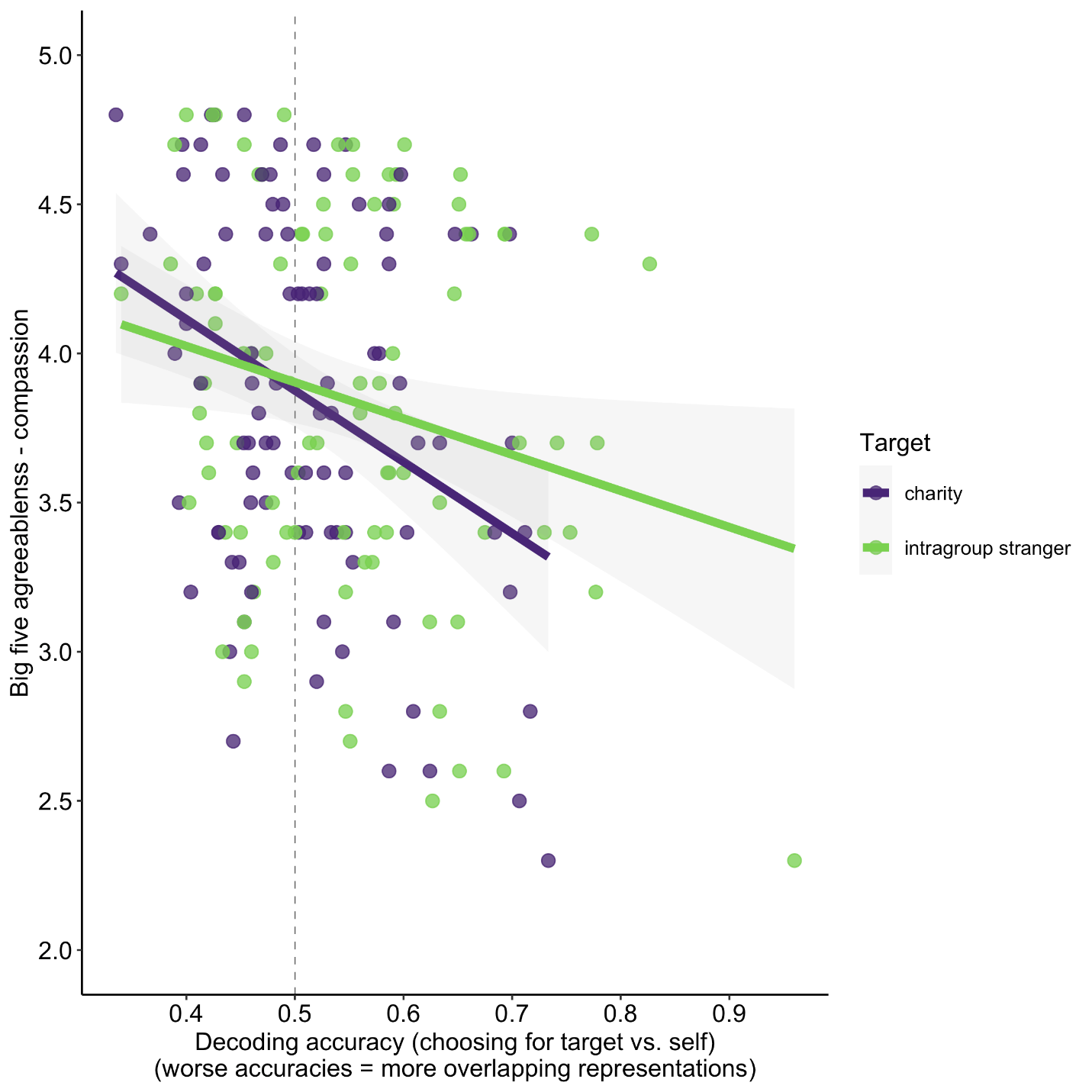


**Figure S19.** Compassion (BFAS) as a function of linear support vector machine decoding or classification accuracy for all participants (no exclusions). Dots are individual participants’ data for charity (purple) and stranger (green) trials. The vertical dashed line indicates chance-level classification accuracy (50%) when decoding whether the target on any given trial was self or other target (charity or stranger). Higher classification accuracies indicate less representational overlap and are associated with reduced compassion at the trait level.

**Deviations from Preregistration**

Study 1 and Study 2 were both preregistered (osf.io/rc4an; osf.io/ncs97). However, we had to deviate very slightly from the preregistered analytic plan. In Study 1, as described in the main text, we changed our effort scaling such that 7 was the most difficult effort level rather than 9, corresponding with reported task demands. This change did not alter our pattern of results (Table S4, S8).

We originally preregistered that we would exclude individuals with less than 80% accuracy in the task. This criterion applied to only 5 participants across our two studies and was relatively arbitrary. Further, low accuracy participants generally reported taking the study seriously in a data quality funnel debriefing question. Therefore, the results and figures in the main text include participants with lower than 80% accuracy. However, analysis excluding participants lower than 80% (Table S16) or lower than 50% accuracy (Table S17), produces nearly identical results.

In both studies, we preregistered predictions that self-reported frequency of donation to charity would be associated with prosocial effort, but we did not discuss these hypotheses in the main text. There was no significant correlation between donation frequency and effortful choice for charity at the participant level (Study 1: r(47) = 0.13, p = .365; Study 2: r(45) = 0.17, p = .251). However, there may be a true effect at the between participant level that we were not sufficiently powered to detect.

| **Term** | **Results** |
| --- | --- |
| (Intercept) | b = 2.31, SE = 0.16, z = 14.77, p < .001, r = 0.54 |
| Effort | b = -1.92, SE = 0.10, z = -19.95, p < .001, r = -0.47 |
| Reward | b = 0.36, SE = 0.10, z = 3.74, p < .001, r = 0.10 |
| Target (Charity) | b = -1.74, SE = 0.08, z = -21.19, p < .001, r = -0.43 |
| Target (Intragroup stranger) | b = -2.61, SE = 0.08, z = -31.34, p < .001, r = -0.58 |
| Effort x Reward | b = -0.04, SE = 0.13, z = -0.27, p = .784, r = -0.01 |
| Effort x Target (Charity) | b = 0.48, SE = 0.11, z = 4.22, p < .001, r = 0.13 |
| Effort x Target (Intragroup stranger) | b = 0.56, SE = 0.11, z = 4.94, p < .001, r = 0.15 |
| Reward x Target (Charity) | b = -0.13, SE = 0.11, z = -1.14, p = .254, r = -0.04 |
| Reward x Target (Intragroup stranger) | b = -0.16, SE = 0.11, z = -1.39, p = .165, r = -0.04 |
| Effort x Reward x Charity | b = 0.07, SE = 0.16, z = 0.41, p = .680, r = 0.02 |
| Effort x Reward x Intragroup stranger | b = -0.004, SE = 0.16, z = -0.02, p = .982, r = 0 |

**Table S16.** Study 2 results with preregistered exclusion criteria including 80% accuracy exclusion. N = 9,774 observations, 45 participants. AIC = 9,250.75. Marginal/Conditional R2 = 0.37/0.51.

| **Term** | **Results** |
| --- | --- |
| (Intercept) | b = 2.31, SE = 0.16, z = 14.77, p < .001, r = 0.54 |
| Effort | b = -1.90, SE = 0.10, z = -19.88, p < .001, r = -0.46 |
| Reward | b = 0.34, SE = 0.10, z = 3.55, p < .001, r = 0.09 |
| Target (Charity) | b = -1.74, SE = 0.08, z = -21.36, p < .001, r = -0.43 |
| Target (Intragroup stranger) | b = -2.61, SE = 0.08, z = -31.62, p < .001, r = -0.58 |
| Effort x Reward | b = -0.01, SE = 0.13, z = -0.08, p = .939, r = 0 |
| Effort x Target (Charity) | b = 0.49, SE = 0.11, z = 4.35, p < .001, r = 0.13 |
| Effort x Target (Intragroup stranger) | b = 0.55, SE = 0.11, z = 4.91, p < .001, r = 0.15 |
| Reward x Target (Charity) | b = -0.12, SE = 0.11, z = -1.09, p = .277, r = -0.03 |
| Reward x Target (Intragroup stranger) | b = -0.13, SE = 0.11, z = -1.16, p = .246, r = -0.04 |
| Effort x Reward x Charity | b = 0.03, SE = 0.16, z = 0.22, p = .829, r = 0.01 |
| Effort x Reward x Intragroup stranger | b = -0.02, SE = 0.16, z = -0.15, p = .881, r = -0.01 |

**Table S17.** Study 2 results with preregistered exclusion criteria and 50% accuracy exclusion. N = 9,981 observations, 46 participants. AIC = 9,447.41. Marginal/Conditional R2 = 0.37/0.50.

**Questionnaires Administered**

We administered a number of questionnaires after the main task. We made no hypotheses about these measures and report no analysis of them in the main text. Below is a table of questionnaires measured and their corresponding column names in the dataset for the potential benefit of other interested researchers (Table S18).

| **Questionnaire name** | **Source** | **Label in Dataset** |
| --- | --- | --- |
| Apathy Motivation Index (social motivation; emotional sensitivity) | Ang et al., 2017 | ami_emoSens_score; ami_socialMot_score |
| Religious Orientation | Lewis et al., 2001 | rz_score |
| Big Five Aspect Scale (compassion, orderliness, industriousness) | DeYoung et al., 2007 | bfas_agree_compassion_score |
| Dispositional Positive Emotion Scale (awe) | Shiota et al., 2006 | awe_score |
| Prosocialness Scale for Adults | Caprara et al., 2005 | psa_score |
| Donation Frequency | How often do you donate to charity? | donate_freq |
| Empathy Index | Jordan et al., 2016 | ei_empathy_score; ei_BC_score |

**Table S18.** Questionnaires measured at baseline, their source, and their corresponding label in the dataset. Add _C for grand-mean centered versions.

**Time-On-Task Effects**

The perceived task demand of a given task is determined not only by the objective demands of the task relative to individual ability, but also by the extent to which an individual is fatigued or motivated at the time of the decision. Thus, effort investment tends to decrease over the course of a study (Lindner et al., 2018). As such, it is possible that decisions to invest effort may shift over the course of the study, as individuals become cognitively fatigued across trials. Further, it is possible that an individuals’ priorities may shift with fatigue in terms of attaining goals for self and others (Hockey, 2013).

We therefore examined the effect of time on task (i.e., trial number) by fitting a generalized mixed-effects model, with effort, reward, target, and trial number and all their two-, three-way, and four-way interactions as regressors (maximal model with participants as a grouping variable). In Study 1, the effect of trial was not significant, b = -0.07, SE = 0.05, z = -1.47, p = .143. That is, overall, participants did not appear to become cognitively fatigued and avoid effort more over time. We also found no significant interaction between target and trial number (ps > .05). However, trial interacted with effort (b = -4.39, SE = 0.85, z = -5.18, p < .001), suggesting that as trial number increased, participants avoided choosing more effortful options much more (Figure S20).

In Study 2, participants chose the effortful option less as the trial number increased, b = -0.117, SE = 0.05, z = -3.53, p < .001. Trial also interacted with target (Figure S21), such that trial number did not affect participants’ choices as much when the target was oneself, but participants became less likely to choose the effortful option when the target was charity (b = -1.89, SE = 0.62, z = -3.02, p = .003) or intragroup stranger (b = -1.16, SE = 0.70, z = -1.65, p = .099), though the latter effect was non-significant.


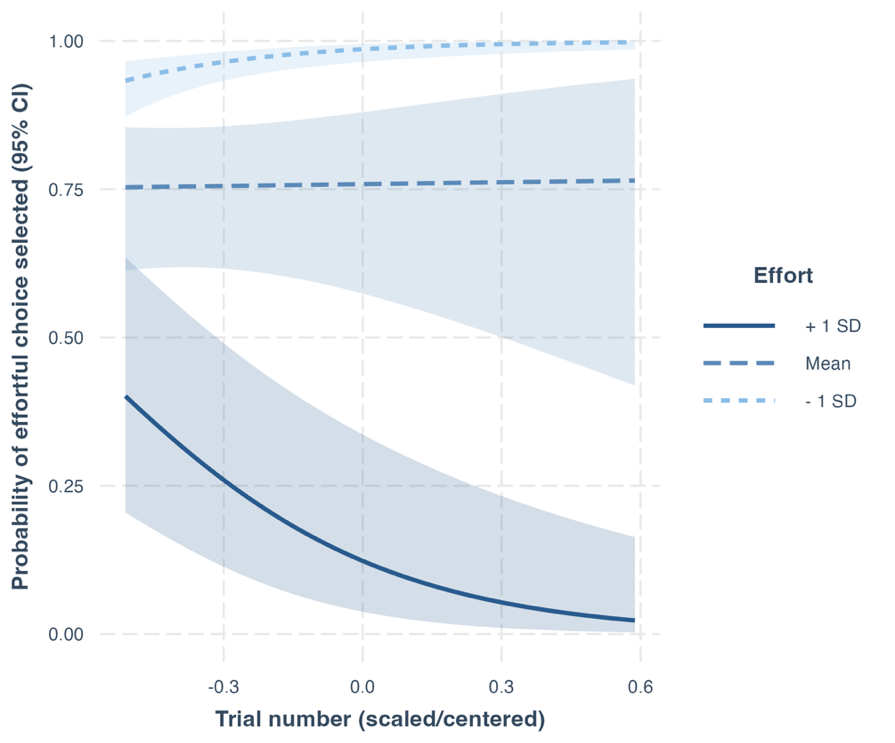


**Figure S20.** Simple effects of trial number for different effort levels in Study 1. Effects are estimates from generalized mixed-effects four-way interaction model predicting choice.

**
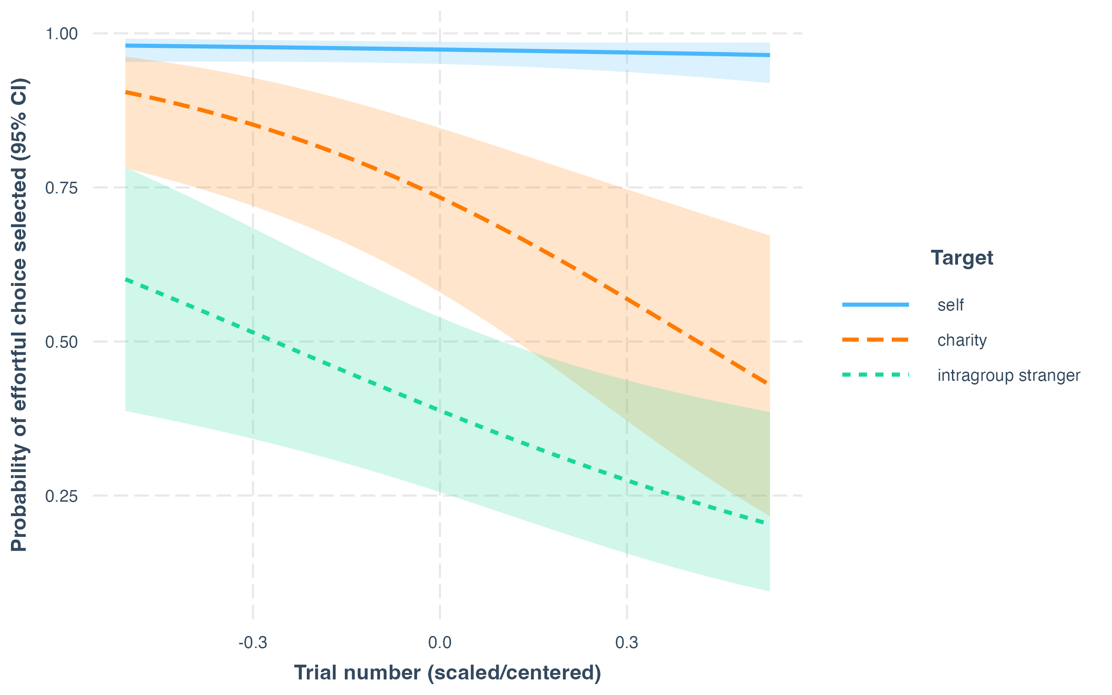
**

**Figure S21.** Simple effects of trial number for different effort levels in Study 2. Effects are estimates from generalized mixed-effects four-way interaction model predicting choice.

**Gender Effects**

We added gender as the fourth predictor to the maximal model reported in the main manuscript (i.e., four-way interaction model). In Study 1, males did not choose the effortful option significantly more than females (b = 0.02, SE = 0.06). However, gender interacted with reward and target to predict choice (Figure S22). The effect of reward on effortful choice was significantly larger for males than females, suggesting that males were more sensitive to the reward manipulation (Figure S22 middle panel). Females, however, were more willing than males to exert effort for their chosen charities (relative to self) (Figure S22 third panel). In Study 2, females were also slightly more willing than males to exert effort for their chosen charities, but this interaction effect was not statistically significant (Figure S23 third panel).

Note that because our studies were underpowered (i.e., relatively few male participants) for detecting gender effects, the above findings should be considered highly exploratory. Further research is needed to investigate how males and females differ in their willingness to invest cognitive effort for self and others, especially when multiple non-self targets are involved.


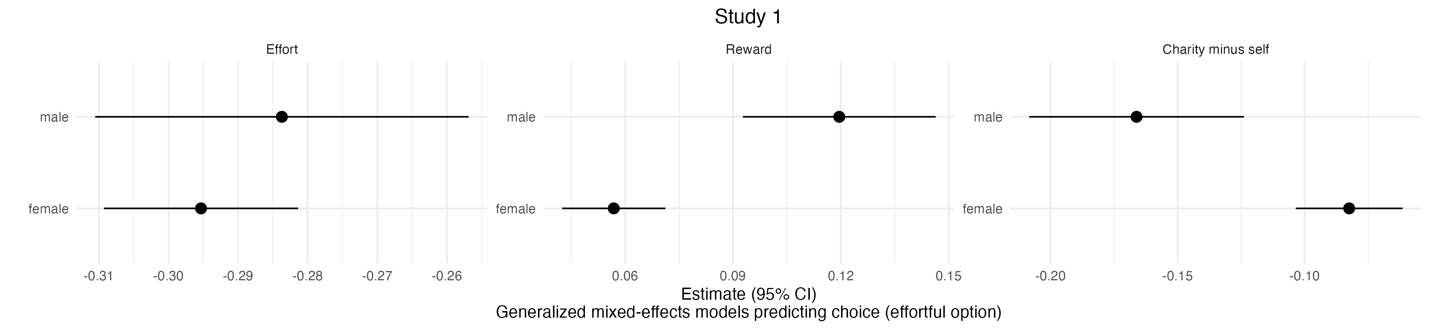
**Figure S22.** Effects of effort, reward, and target for females and males in Study 1. Effects are estimates from generalized mixed-effects four-way interaction model predicting choice.


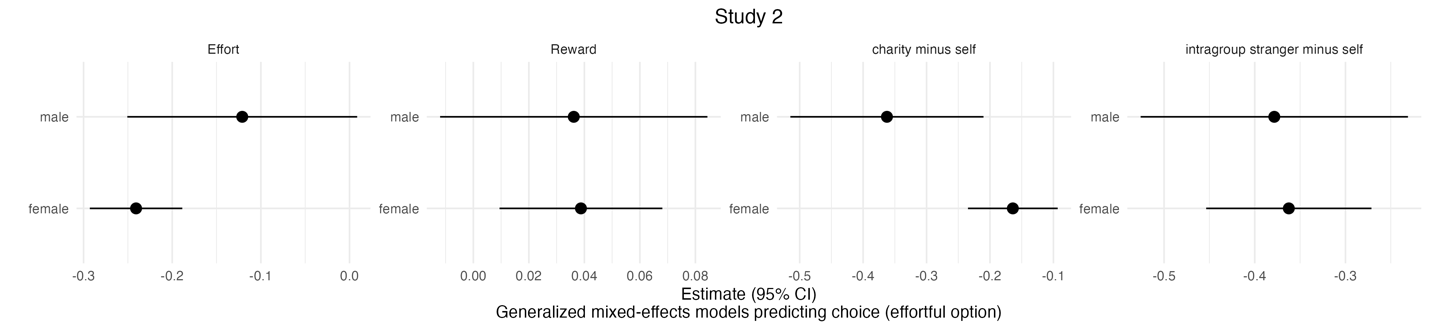
**Figure S23.** Effects of effort, reward, and target for females and males in Study 2. Effects are estimates from generalized mixed-effects four-way interaction model predicting choice.

**Effects of Anticipated Task Performance (Prior Trial Accuracy)**

Individuals’ willingness to invest effort can be influenced by their anticipated task performance (Westbrook et al., 2019). Thus, we examined whether effortful choice might be influenced by the previous trial’s task accuracy by adding this variable as a fourth predictor to the maximal model reported in the main manuscript (i.e., four-way interaction model). In Study 1, prior trial accuracy was not significantly associated with choice, b = -0.03, SE = 0.03, z = -0.98, p = .328. It also did not interact with any other predictors in the model (ps > .204). The results were similar in Study 2 where prior trial accuracy was not significantly associated with choice, b = -0.12, SE = 0.15, z = -0.76, p = .448.

**Choice Time Effects**

On each trial, participants had up to 5 seconds to choose between the baseline and effortful options. It is possible that differences in the speed at which participants made these decisions may be associated with differences in effortful choice. For example, participants may take this time to engage in perspective taking with the target of a given trial, which may predict engaging in effort on their behalf. On the other hand, longer decision times may reflect participant hesitation in the face of a questionable trade-off, and therefore longer decision times may predict engaging in the baseline option. Further, effort choice time may show different associations when decided on behalf of different targets.

First, we looked at the variables that influenced choice time. We modeled single-trial choice times using a linear mixed-effects model, with effort, reward, and target and all their two- and three-way interactions as regressors (maximal model with participants as a grouping variable). Participants took more time to decide as effort increased (Study 1: b = 0.06, SE = 0.01, z = 5.95, p < .001; Study 2: b = 0.05, SE = 0.01, z = 6.96, p < .001), and they took longer when making choices for charity (Study 1: b = 0.03, SE = 0.01, t = 2.09, p = .036; Study 2 charity vs self: b = 0.08, SE = 0.01, z = 6.18, p < .001) or strangers, (Study 2 intragroup stranger vs self: b = 0.15, SE = 0.01, z = 11.14, p < .001) than when choosing for the self.

Next, we added choice time to the maximal model reported in the main text. We modeled single-trial choice using a generalized mixed-effects model, with effort, reward, target, and choice time and all their two-, three-way, and four-way interactions as regressors (maximal model with participants as a grouping variable). In Study 1, choice time was not a significant predictor of choice, b = -0.01, SE = 0.01, z = -0.80, p = .422, but it interacted with reward, b = 0.25, SE = 0.10, z = 2.62, p = .009. That is, when more reward was offered, longer choice times were associated with increased likelihood of choosing the effortful option (Figure S24).

In Study 2, choice time was a significant predictor of choice, b = -0.01, SE = 0.006, z = -2.09, p = .037, and, as with Study 1, choice time interacted with reward, b = 0.54, SE = 0.17, z = 3.14, p = .002 (Figure S25). We also found a choice time-target interaction (Figure S26): Longer choice times were associated with avoiding effort when making decisions for oneself, b = -0.39, SE = 0.12, z = -3.27, p < .001, but not charity, b = 0.03, SE = 0.08, z = 0.38, p = .71, or intragroup stranger, b = 0.03, SE = 0.06, z = 0.53, p = .60.

While speculative, these results may suggest that longer effortful choice times in Study 2 when deciding to earn rewards for the self may be indicative of hesitation, and ultimately be associated with avoiding the effortful response. In the current study, participants typically made their decisions well within the 5 second limit. Future research should explore how varying time pressure influences decisions to invest cognitive effort for self and others (Teoh et al., 2020).


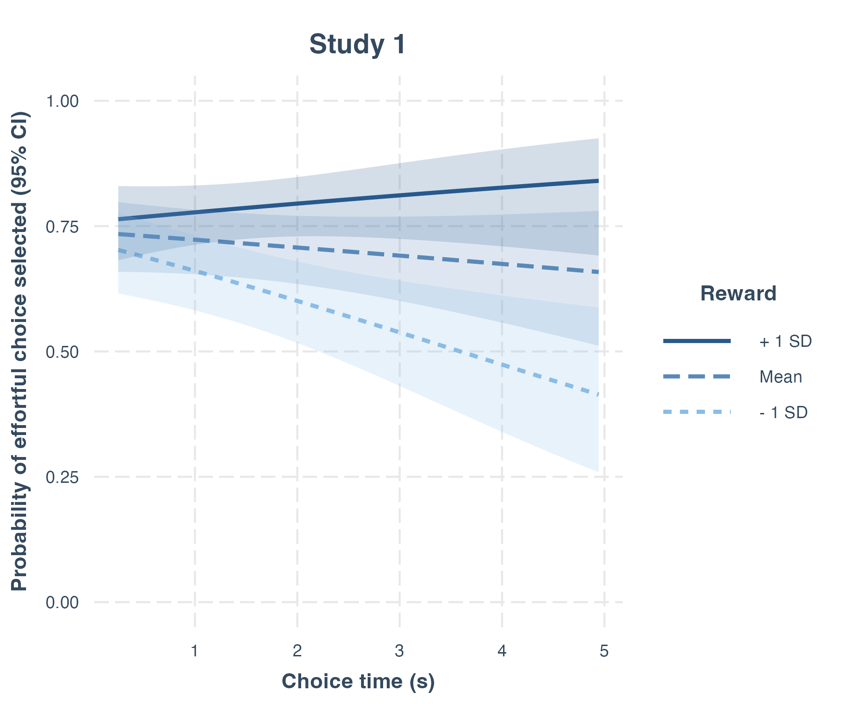


**Figure S24.** Simple effects of choice time for different reward levels in Study 1.


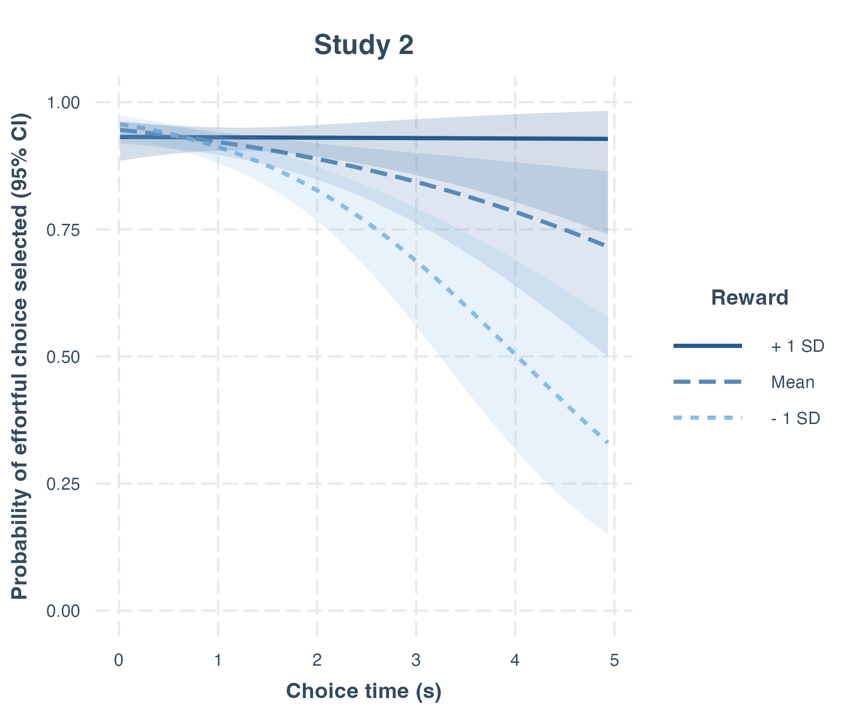


**Figure S25.** Simple effects of choice time for different reward levels in Study 2.


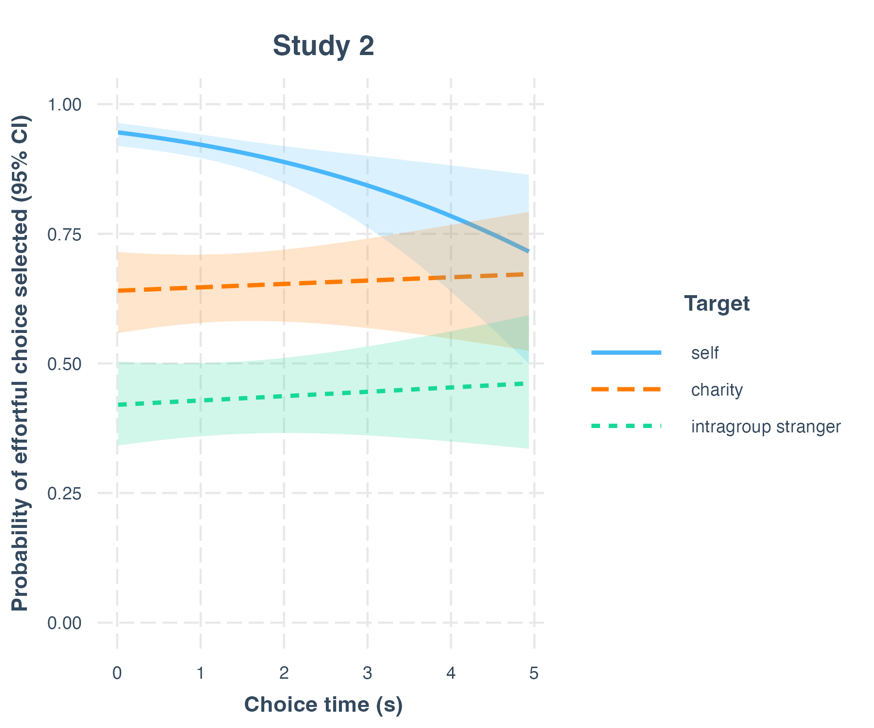


**Figure S26.** Simple effects of choice time for different targets in Study 2.

**Industriousness and Cognitive Effort**

We explored whether willingness to invest cognitive effort was associated with the industriousness facet of the Big 5 (Fong & Tosi, 2007). We found that industriousness did not correlate with overall proportion of effortful options chosen in both studies (Study 1: r(47) = -0.18, p = .225; Study 2: r(45) = 0.005, p = .974). However, our study was powered to detect only within-person effects, so these non-significant effects could reflect the lack of statistical power to detect between-subject correlations/effects.

**Open Science Materials**

For materials, data, and code see: osf.io/verhb/

Study 1 preregistration: osf.io/rc4an

Study 2 preregistration: osf.io/ncs97

**References**

DeYoung, C. G., Quilty, L. C., & Peterson, J. B. (2007). Between facets and domains: 10 aspects of the Big Five. *Journal of Personality and Social Psychology*, *93*(5), 880–896. https://doi.org/10.1037/0022-3514.93.5.880

Fong, E. A., & Tosi Jr., H. L. (2007). Effort, performance, and conscientiousness: An agency theory perspective. *Journal of Management*, *33*(2), 161–179. https://doi.org/10.1177/0149206306298658

Hockey, R. (2013). *The Psychology of Fatigue: Work, Effort and Control*. Cambridge University Press. https://doi.org/10.1017/CBO9781139015394

Lindner, C., Nagy, G., & Retelsdorf, J. (2018). The need for self-control in achievement tests: Changes in students’ state self-control capacity and effort investment. *Social Psychology of Education*, *21*(5), 1113–1131. https://doi.org/10.1007/s11218-018-9455-9

Lockwood, P. L., Hamonet, M., Zhang, S. H., Ratnavel, A., Salmony, F. U., Husain, M., & Apps, M. A. J. (2017). Prosocial apathy for helping others when effort is required. *Nature Human Behaviour*, *1*(7), 1–10. https://doi.org/10.1038/s41562-017-0131

Mantel, N. (1967). The Detection of Disease Clustering and a Generalized Regression Approach. *Cancer Research*, *27*(2 Part 1).

Meyer, M. L., & Lieberman, M. D. (2018). Why people are always thinking about themselves: Medial prefrontal cortex activity during rest primes self-referential processing. *Journal of Cognitive Neuroscience*, *30*(5), 714–721. https://doi.org/10.1162/jocn_a_01232

Tamir, D. I., & Mitchell, J. P. (2010). Neural correlates of anchoring-and-adjustment during mentalizing. *Proceedings of the National Academy of Sciences of the United States of America*, *107*(24), 10827–10832. https://doi.org/10.1073/pnas.1003242107

Teoh, Y. Y., Yao, Z., Cunningham, W. A., & Hutcherson, C. A. (2020). Attentional priorities drive effects of time pressure on altruistic choice. *Nature Communications*, *11*(1), 3534. https://doi.org/10.1038/s41467-020-17326-x

Westbrook, A., Lamichhane, B., & Braver, T. (2019). The subjective value of cognitive effort is encoded by a domain-general valuation network. *Journal of Neuroscience*, *39*(20), 3934–3947. https://doi.org/10.1523/JNEUROSCI.3071-18.2019
